# A Macro-Anchored Physics-Informed GAN for RUL Prediction Under Continuous Block Missing Data

**DOI:** 10.3390/s26196051

**Published:** 2026-09-24

**Authors:** Yongkang Peng, Jianxun Zhang, Zhengxin Zhang, Xiaosheng Si, Dangbo Du

**Affiliations:** Rocket Force University of Engineering, Xi’an 710025, China; dq00ak@163.com (Y.P.); jx-zhang14@tsinghua.org.cn (J.Z.); zhangzx.2006@tsinghua.org.cn (Z.Z.); sixiaosheng@126.com (X.S.)

**Keywords:** physics-informed GAN, continuous block-missing time series, temporal degradation trajectory reconstruction, remaining useful life prediction, uncertainty-aware prognostics

## Abstract

Continuous block missingness in condition monitoring data poses a major challenge to the reliability of Remaining Useful Life (RUL) prediction for industrial equipment. Existing frameworks, including direct prediction from incomplete observations and decoupled two-stage imputation–prediction pipelines, suffer from a noticeable performance decline under prolonged data voids. Although Generative Adversarial Network (GAN)-based generative models have shown promise in data repair, they remain limited by dimensionality contamination from fixed-dimension architectures, purely data-driven over-smoothing that does not explicitly account for physical degradation laws, the difficulty of capturing instance-specific heterogeneous degradation signatures, and limited characterization of downstream predictive epistemic uncertainty under reconstructed inputs. To address these limitations, we propose a two-stage reconstruction–prognosis framework with uncertainty-aware downstream prediction, integrating two core modules: a Macro-Anchored Physics-Informed Generative Adversarial Network (MAP-GAN) tailored for missing sequence reconstruction, and an Attention-Bidirectional Long Short-Term Memory network integrated with Monte Carlo Dropout (Attention-BiLSTM-MCD) for uncertainty-aware prognosis, which provides an empirical estimate of predictive uncertainty under reconstructed inputs and produces RUL estimates with probabilistic intervals. Specifically, a macro-anchored micro-window generator confines the receptive field to mitigate dimensionality contamination; degradation-informed physical constraints are embedded into the adversarial objective to encourage the synthesized trajectories to satisfy physical degradation constraints; an encoder-guided latent space inversion mechanism adaptively captures individual-specific degradation patterns; and the Attention-BiLSTM-MCD approximates epistemic uncertainty to support prognostic reliability. The effectiveness of the proposed method is examined on a lithium-ion battery dataset with continuous block missingness, and is further verified through missing-pattern sensitivity analyses (missing ratios of 20–60% and early/late missing positions), physics-constraint term-wise ablation with weight sensitivity, an uncertainty-calibration analysis, and an external validation on a second, independent battery dataset.

## 1. Introduction

Driven by the deep integration of Industry 4.0 and the Industrial Internet of Things (IIoT), modern industrial equipment is undergoing a rapid paradigm shift toward automation, digitalization, and intelligent operation, which has substantially elevated system complexity and functional integration [1]. However, such engineered systems inevitably experience performance degradation throughout their service life [2]. In industrial practice, equipment degradation seldom presents as an abrupt failure; rather, it evolves progressively and cumulatively, driven by the coupled effects of complex physicochemical and operational factors—including material micro-damage (e.g., fatigue, wear, creep, corrosion), extreme or off-design operating conditions, environmental erosion agents, manufacturing defects, and sensor or control system failures exhibiting distinct evolutionary regularity [3]. By uncovering this inherent evolutionary regularity, accurate Remaining Useful Life (RUL) prediction serves as a core enabling technology within the Prognostics and Health Management (PHM) framework. It enables proactive condition-based maintenance, helps prevent catastrophic equipment failures, and reduces unscheduled downtime and corresponding economic losses [4]. As a result, RUL prediction has attracted increasing attention from both academic and industrial communities, such as aero-engines [5], lithium-ion batteries [6], and rolling bearings [7].

Existing approaches for RUL prediction are generally divided into two categories: physics-based models and data-driven methods. Physics-based models rely on clear physical failure mechanisms, so they have high interpretability and reliable prediction accuracy. However, establishing accurate mathematical formulations for such models is often impractical for highly integrated modern industrial equipment. Data-driven methods get around this constraint by directly extracting degradation features from condition monitoring (CM) data. Driven by the large-scale growth of industrial sensor data, deep learning has quickly become a dominant method for RUL prognostics. Its key advantage is that it can automatically learn complex nonlinear degradation trajectories without needing explicit physical prior knowledge. In recent years, a wide range of deep neural network architectures, such as convolutional neural networks (CNN) [8], recurrent neural networks (RNN) [9], Long Short-Term Memory networks (LSTM) [10], temporal convolutional networks (TCN) [11], and hybrid deep learning approaches [12], have been employed for RUL prediction. It is worth noting that the theoretical performance and real-world applicability of most conventional deep learning architectures are constrained by an idealized assumption: the CM data used for model training and inference are perfectly continuous, regularly sampled temporal sequences.

However, this idealized assumption is hard to satisfy in real-world industrial scenarios [13]. Ubiquitous operational anomalies across industrial scenarios (e.g., sensor faults, communication bus contention, periodic system sleep modes, and transient data transmission failures) frequently break the continuity of observation sequences, resulting in severe data fragmentation, formally defined herein as continuous block missingness (hereinafter referred to as block missingness). Recently, recovering such severe structural discontinuities has emerged as a critical bottleneck across diverse engineering domains, ranging from bridge health monitoring [14] to power dispatch networks [15]. Unlike the isolated, random point dropouts adopted in most traditional imputation studies, block missingness appears as long-term observation gaps that cover dozens or even large numbers of consecutive operating cycles. This essential difference in temporal structure redefines the data recovery problem and greatly raises its difficulty. Isolated point dropouts can be reliably restored through local mathematical interpolation, yet continuous block missingness causes serious structural discontinuities in the time dimension, which renders traditional point-wise imputation methods less capable of supporting subsequent degradation modeling and RUL prediction.

Existing RUL prediction methods for incomplete data generally follow two routes: direct mapping or a decoupled two-stage pipeline. Direct mapping strategies skip data repair and directly estimate RUL from incomplete inputs, using models such as improved Hidden Markov Models [16], recurrent embeddings [17,18], tree-based ensembles [19,20], and graph attention networks [21]. These methods perform well for random point missingness, but have difficulty maintaining temporal consistency when dealing with continuous block missingness. In contrast, the widely used two-stage approach first imputes missing data before building prognostic models. While recent advancements have attempted to tackle continuous data gaps via hybrid signal decomposition with deterministic deep sequence models [14] or transfer learning [15], these general-purpose recovery techniques, alongside traditional imputation methods—including statistical interpolation [22], nearest-neighbor matching [23], and Hot Deck imputation [24]—mainly optimize numerical reconstruction accuracy, while the physical consistency of degradation trajectories is not explicitly considered. This emphasis on numerical smoothness tends to attenuate important high-frequency fluctuations. More importantly, the two-stage process is subject to error accumulation: small errors from imputation may be amplified by later prognostic models, which can reduce the final prediction accuracy [25].

Beyond the main limitations of the above two mainstream RUL prediction frameworks under missing data, Generative Adversarial Networks (GANs), as representative deep generative models, have been widely applied to RUL prediction under missing data scenarios in recent years. Benefiting from their strong capability in fitting high-dimensional temporal data distributions and generating complex nonlinear degradation trajectories, GANs have become one of the prevailing technical routes for tackling data missing challenges in industrial PHM. For example, Lee et al. [26] proposed a GAN-based framework, constructing a generator and a predictor via deep neural networks (DNNs) to simultaneously realize missing value estimation and fault prediction for Train Control and Management System data. Representative adversarial frameworks for missing-data imputation and time-series generation, GAIN [27] and TimeGAN [28], respectively, formalized these problems in a generative-adversarial manner; however, such frameworks are primarily designed for scattered point missingness or unconditional sequence generation, and their fixed-dimension decoders are not directly amenable to long contiguous gaps embedded in full-lifecycle degradation trajectories. Relevant studies have examined data-driven prognostic and data-repair methods in typical industrial systems, including aero-engines [29], lithium-ion batteries [30], and bearings [31], demonstrating their effectiveness under the evaluated conditions.

Nevertheless, conventional GAN models exhibit inherent limitations for RUL prediction under industrial continuous block missingness scenarios, failing to fully overcome the aforementioned limitations of the two mainstream frameworks, which motivates this study. First, their fixed-dimension architecture struggles to adapt to variable-length full-lifecycle degradation trajectories: zero-padding for uneven-length CM sequences introduces dimensionality contamination—defined herein as the introduction of non-physical artificial artifacts caused by forced zero-padding or arbitrary truncation, which propagate through latent spaces and distort the underlying dynamic representations [32]—while modeling continuous full-lifecycle sequences with deep recurrent structures faces gradient vanishing, which limits the capture of critical local degradation micro-dynamics or long-range dependencies. Second, their adversarial objective only minimizes the statistical distance between generated and real samples, which can lead to a “regression-to-the-mean” tendency of mathematical smoothness. Without explicit degradation-informed priors, the synthesized trajectories may deviate from physically valid degradation manifolds and the real degradation laws [33]. Third, population-level generative models tend to generate overly smoothed estimates, which limits their ability to adaptively capture the highly heterogeneous, equipment-specific degradation features that are important for accurate RUL prediction [34]. Finally, most methods follow a decoupled two-stage pipeline. They tend to neglect the epistemic noise introduced in the generative process and directly regard the imputed sequences as ground truth. This leaves the downstream predictive epistemic uncertainty under reconstructed inputs largely uncharacterized, resulting in deterministic RUL point estimates that provide limited probabilistic information for predictive maintenance.

Before detailing the proposed method, it is necessary to delimit its position with respect to the closest technical routes. (i) Compared with adversarial frameworks for missing-data imputation or time-series generation, such as GAIN [27] and TimeGAN [28], respectively, the proposed generator restricts generation to lifecycle-stage-conditioned micro-windows, and online repair is performed by latent inversion within a frozen pre-trained manifold rather than by direct decoding from observed coordinates; this yields instance-specific reconstructions instead of population-averaged estimates. (ii) Compared with classical physics-informed neural networks (PINNs) [35], which typically encode explicit governing equations (e.g., electrochemical partial differential equations) to be embedded in the training objective, this work does not assume any closed-form mechanistic model. Instead, empirical degradation regularities—capacity amplitude bounds, fade-rate and curvature consistency, and fluctuation preservation—are distilled into a soft physical regularization of the adversarial objective, which remains applicable when mechanistic models are unavailable or poorly identified. (iii) Compared with modern deep imputation models, including BRITS [36], SAITS [37], the diffusion-based CSDI [38], and the probabilistic GP-VAE [39], which are typically trained and evaluated under randomly scattered missing patterns, the proposed framework explicitly targets continuous block missingness and couples trajectory reconstruction with downstream uncertainty-aware RUL prediction. These distinctions define the methodological boundary of this study.

To address these limitations, this study proposes a Macro-Anchored Physics-Informed Generative Adversarial Network (MAP-GAN). It is a two-stage reconstruction–prognosis framework with uncertainty-aware downstream prediction, designed for instance-level missing sequence reconstruction and uncertainty-aware RUL prediction. Unlike mainstream methods that treat block missingness only as a pure mathematical data-filling problem, MAP-GAN reformulates imputation as a degradation trajectory reconstruction problem guided by the inherent failure evolution laws of equipment. The main contributions are as follows:

(1) To reduce the influence of zero-padding artifacts and alleviate the long-range dependency limits of fixed-dimension architectures, we constrain the generative process within a localized optimal receptive field. By anchoring micro temporal windows to macroscopic degradation stages, MAP-GAN supports accurate reconstruction of local degradation dynamics with reduced dimensionality contamination. Unlike generic conditional GANs that condition on exogenous attributes, the anchor index encodes the relative position of each micro-window within the entire degradation lifecycle, so the conditioning variable itself carries a physical, stage-dependent prior.
(2) To mitigate the “regression-to-the-mean” tendency of purely data-driven imputation that over-pursues mathematical smoothness, degradation-informed physical constraints (amplitude bounds, shape derivative constraints, fluctuation variance penalties) are embedded into the adversarial objective, encouraging the synthesized trajectories to satisfy physical degradation constraints. These constraints act as soft physical regularization distilled from empirical degradation laws rather than as governing-equation penalties of classical PINNs, and their individual contributions are quantified through dedicated term-wise ablation and weight sensitivity analyses in Section 4.
(3) To alleviate the over-smoothing effect of population-level generative models, we propose an encoder-guided latent space inversion mechanism. It uses a lightweight encoder to rapidly map fragmented observations into an initial latent representation, and further refines it within the frozen pre-trained latent space. This mechanism adaptively captures individual-specific degradation patterns, improving the fidelity and heterogeneity of the degradation trajectory reconstruction. In this sense, online reconstruction is formulated as an instance-specific inversion problem rather than a population-level generation process.
(4) To quantify predictive epistemic uncertainty under reconstructed inputs, the framework feeds the reconstructed sequences into an attention-augmented bidirectional LSTM (BiLSTM) network embedded with Monte Carlo Dropout (MCD). Rather than producing a single deterministic point prediction as in conventional two-stage schemes, this module provides an empirical estimate of the downstream predictive epistemic uncertainty conditional on the reconstructed input, together with uncertainty-aware RUL estimates with probabilistic intervals, offering potential support for predictive maintenance applications.

The remainder of this paper is organized as follows: Section 2 elaborates on the problem formulation; Section 3 introduces the proposed methodology, with the offline prior knowledge acquisition and the online instance-level deployment; Section 4 examines the method’s efficacy through comparative experiments; and Section 5 summarizes the research contributions.

## 2. Problem Formulation

This section formally defines the dataset structure and observational topology of continuous block missing CM data in practical industrial scenarios, clarifies the core boundaries of the targeted RUL prediction problem, and provides a theoretical basis and problem constraints for subsequent model design and methodology development.

### 2.1. Dataset Definition and Observational Topology

We formally define two core datasets for offline training and online inference, respectively.

(1) Complete Historical Dataset:

Let $D_{\mathrm{hist}}={\left\{\left(X_{i}^{\mathrm{hist}},y_{i}^{\mathrm{hist}}\right)\right\}}_{i=1}^{N}$ denote the complete offline historical dataset, where *N* is the total number of run-to-failure equipment units. For the *i*-th unit, $X_{i}^{\mathrm{hist}}={\left[x_{i,1}^{\mathrm{hist}},x_{i,2}^{\mathrm{hist}},\ldots ,x_{i,T_{i}}^{\mathrm{hist}}\right]}^{⊤}\in R^{T_{i}}$ represents the uninterrupted full-lifecycle capacity degradation sequence, with $T_{i}$ denoting the maximum operational cycle length of the unit. The corresponding cycle-wise ground-truth RUL labels are collected as $y_{i}^{\mathrm{hist}}={\left[y_{i,1}^{\mathrm{hist}},y_{i,2}^{\mathrm{hist}},\ldots ,y_{i,T_{i}}^{\mathrm{hist}}\right]}^{⊤}\in R_{+}^{T_{i}}$. This dataset contains no missing values and serves as the empirical basis for learning the degradation-informed generative priors and training the downstream RUL predictor.

(2) Incomplete Online Monitoring Dataset:

Let $D_{\mathrm{online}}={\left\{\left(X_{j}^{\mathrm{obs}},M_{j}\right)\right\}}_{j=1}^{N_{\mathrm{online}}}$ denote the online monitoring dataset collected from $N_{\mathrm{online}}$ in-service equipment units. Define $X_{j}^{\mathrm{true}}\in R^{T_{j}}$ as the underlying noise-free physical degradation sequence, which characterizes the intrinsic aging trend of equipment without sensor measurement error. Considering inevitable industrial sensing noise, the fully sampled sensor output is denoted as $X_{j}^{\mathrm{online}}$, which represents the complete noisy baseline signal under normal communication conditions. To describe the widespread continuous data loss in industrial scenarios, a binary masking vector is defined as $M_{j}={\left[m_{j,1},m_{j,2},\ldots ,m_{j,T_{j}}\right]}^{⊤}\in {\left\{0,1\right\}}^{T_{j}}$, where $m_{j,t}=1$ denotes valid measurement acquisition at cycle *t*, and $m_{j,t}=0$ indicates data loss caused by transmission interruption or sensor failure. The associated index sets are $\Omega_{j}^{\mathrm{obs}}=\left\{t:m_{j,t}=1\right\}$ and $\Omega_{j}^{\mathrm{miss}}=\left\{t:m_{j,t}=0\right\}$. Different from the ideal MCAR point missingness in most existing studies, invalid entries in $M_{j}$ appear as continuous block gaps.

By applying the masking vector to the complete noisy sensing sequence $X_{j}^{\mathrm{online}}$, the available observed part and the unmeasured missing part are decoupled via the Hadamard product (⊙):

(1) $$X_{j}^{\mathrm{obs}}=X_{j}^{\mathrm{online}}⊙M_{j}$$

(2) $$X_{j}^{\mathrm{miss}}=X_{j}^{\mathrm{online}}⊙\left(1-M_{j}\right)$$

where 1 is an all-one vector of the same dimension. In this setting, $X_{j}^{\mathrm{true}}$ serves as the physical degradation ground truth, $X_{j}^{\mathrm{online}}$ represents the complete noisy sensor sequence, and $X_{j}^{\mathrm{obs}}$ is the fragmented online input finally used for inference. $X_{j}^{\mathrm{true}}$ is a conceptual latent degradation process that is not directly observed; in the experiments of Section 4, the reconstruction quality is evaluated against the held-out measured entries of $X_{j}^{\mathrm{online}}$. Given the fragmented observation $X_{j}^{\mathrm{obs}}$, the proposed framework reconstructs the missing segment $X_{j}^{\mathrm{miss}}$, encourages consistency of the reconstructed sequence with the underlying degradation trajectory $X_{j}^{\mathrm{true}}$, and subsequently estimates the probabilistic RUL distribution of the industrial equipment.

### 2.2. Core Problem Boundaries

Based on the formalized continuous block-missing observational topology, high-precision and reliable RUL prediction is transformed into a cascaded challenge of ill-posed inverse feature recovery and downstream predictive uncertainty quantification. In this section, we formalize the two core sub-problems and clarify their mathematical boundaries.

(1) Ill-Posed Sequence Reconstruction Problem:

Severe continuous block missingness masks the underlying nonlinear degradation dynamics of industrial equipment. Traditional imputation paradigms are limited in this scenario, as they formulate the objective merely as minimizing localized numerical errors over the online unit *j*:

(3) $$\min∥{\hat{X}}_{j}-X_{j}^{\mathrm{true}}∥$$

where ${\hat{X}}_{j}\in R^{T_{j}}$ denotes the reconstructed complete sequence of the online unit *j*, consistent with the notation defined in Section 2.1. These methods implicitly treat time-series points as independent observations, without explicitly considering the global degradation process and the irreversible degradation constraints of industrial equipment.

Accordingly, we redefine the mathematical boundary of the reconstruction problem: instead of simple numerical interpolation, the core objective is to recover the underlying temporal distribution of degradation from the masked observation $X_{j}^{\mathrm{obs}}$. Specifically, we aim to learn a generative mapping function F, such that the reconstructed sequence ${\hat{X}}_{j}=F\left(X_{j}^{\mathrm{obs}},M_{j}\right)$ resides close to the manifold of physically valid degradation trajectories $M_{\mathrm{phys}}$. To encourage ${\hat{X}}_{j}\in M_{\mathrm{phys}}$, the objective function constrains not only the absolute amplitude of the sequence (${\hat{x}}_{j,t}$), but also the dynamic temporal consistency, including the first-order degradation rate ($\Delta {\hat{x}}_{j,t}={\hat{x}}_{j,t+1}-{\hat{x}}_{j,t}$) and the second-order degradation curvature ($\Delta^{2}{\hat{x}}_{j,t}=\Delta {\hat{x}}_{j,t+1}-\Delta {\hat{x}}_{j,t}$).

The mask $M_{j}$ is kept fully general in this formulation: any contiguous block configuration, including blocks that touch either end of the observation window, is admissible. The main experimental protocol of this study (Section 4.1) adopts an interior block surrounded by bidirectional context, which corresponds to the practically common retrospective or delayed online reconstruction scenario where measurements after the missing block have already become available before RUL inference. One-sided configurations (blocks starting at the beginning or ending at the boundary of the window) are additionally evaluated as stress tests in the missing-position sensitivity analysis (Section 4.5), where the reconstruction degrades gracefully from interpolation toward extrapolation. Strictly causal real-time forecasting during an ongoing data outage is a distinct prediction problem that is not the focus of this study.

(2) Uncertainty-Aware Prognostic Inference:

Traditional RUL prediction typically formulates the problem as learning a deterministic point-mapping function, which can be formulated as:

(4) $$f_{\theta }:X_{i,t-W+1:t}^{\mathrm{hist}}\mapsto y_{i,t}^{\mathrm{hist}},\;f_{\theta }:R^{W}\to R_{+}$$

where $X_{i,t-W+1:t}^{\mathrm{hist}}$ denotes a fully observed historical degradation window of length *W*, $y_{i,t}^{\mathrm{hist}}$ is the corresponding cycle-wise RUL label, and θ collects the trainable predictor parameters. While this direct mapping achieves acceptable performance on the idealized and complete dataset $D_{\mathrm{hist}}$, it exhibits limitations under extreme data missingness. When the input sequence ${\hat{X}}_{j}$ contains synthetically reconstructed segments, inherent generative noise and epistemic uncertainty are introduced. If a deterministic model directly processes this reconstructed data, the imputation uncertainty may propagate and be amplified through the deep neural network layers, ultimately resulting in overconfident yet less reliable RUL point estimates.

To address this structural limitation, we reformulate the RUL prediction problem as a probabilistic Bayesian inference problem. Instead of relying on a deterministic mapping, obtaining the predictive distribution requires marginalizing over both the imputation posterior $p({\hat{X}}_{j}|X_{j}^{\mathrm{obs}})$ and the prognostic model’s parameter posterior $p(\theta |D_{\mathrm{hist}})$:

(5) $$p\left({\hat{y}}_{j,t}|X_{j}^{\mathrm{obs}},D_{\mathrm{hist}}\right)=\;\int \int \;p\left({\hat{y}}_{j,t}|{\hat{X}}_{j},\theta \right)p\left({\hat{X}}_{j}|X_{j}^{\mathrm{obs}}\right)p\left(\theta |D_{\mathrm{hist}}\right)\;d{\hat{X}}_{j}\;d\theta$$

Equation (5) constitutes the ideal full uncertainty decomposition, in which both the imputation posterior $p({\hat{X}}_{j}|X_{j}^{\mathrm{obs}})$ and the predictor-parameter posterior $p(\theta |D_{\mathrm{hist}})$ are marginalized. The implementation adopted in this study approximates the conditional form $p\left({\hat{y}}_{j,t}|{\hat{X}}_{j},D_{\mathrm{hist}}\right)=\int p\left({\hat{y}}_{j,t}|{\hat{X}}_{j},\theta \right)p\left(\theta |D_{\mathrm{hist}}\right)\;d\theta$ via Monte Carlo Dropout: a single deterministic reconstructed sequence is used, and the reported intervals quantify the predictive epistemic uncertainty of the predictor conditional on that reconstruction, rather than an explicit propagation of the imputation posterior. The gap between the ideal decomposition and this conditional approximation is examined empirically in Section 4.4. Ultimately, the framework outputs two core prognostic characteristics: the predictive mean $E[{\hat{y}}_{j,t}|{\hat{X}}_{j}]$ as the RUL point estimate and the predictive variance $\mathrm{Var}({\hat{y}}_{j,t}|{\hat{X}}_{j})$, which supports probabilistic interval construction for maintenance decisions.

## 3. Methodology

To address the Ill-Posed Sequence Reconstruction Problem and the Uncertainty-Aware Prognostic Inference challenge formulated in Section 2, this section details the operational architecture of the proposed MAP-GAN.

### 3.1. Overall Framework Structure

To bridge the gap between theoretical physical degradation modeling and online industrial deployment, the MAP-GAN framework decouples the full prognostic procedure into two sequential phases: the offline prior knowledge acquisition phase and the online instance-level deployment phase. Centered on the macro-anchored micro-window architecture, this dual-phase pipeline is implemented through four modules, as illustrated in Figure 1. The workflow of each phase is detailed below.

(1) Phase I: Offline Prior Knowledge Acquisition
  Instead of learning arbitrary variable-length full-lifecycle trajectories, the offline phase extracts generalized, degradation-informed priors from the complete historical dataset $D_{\mathrm{hist}}$ using fixed-length micro-window segments. This design reduces the need for zero-padding and alleviates long-range dependency issues. Two parallel training procedures are implemented:
  1) Physics-Informed MAP-GAN Pre-training. To learn the intrinsic degradation manifolds, the macro-anchored generator and discriminator are adversarially trained on the complete dataset. Degradation-informed physical constraints are embedded into the objective function to encourage the generative model to acquire generalized, physically consistent capacity degradation laws with reduced dimensionality contamination.
  2) Prognostic Benchmark Establishment. We pre-train an Attention-BiLSTM network using the complete historical degradation sequences. This builds a stable mapping from high-fidelity degradation features to ground-truth RUL labels, forming a baseline prognostic model for online inference.
(2) Phase II: Online Instance-Level Deployment
  During equipment operation, the framework switches to online inference. It focuses on repairing continuous block missingness in real-time sequences, while separating generative noise from the dominant physical degradation trend. This phase includes two successive core steps:
  1) Adaptive Latent Inversion for Trajectory Reconstruction. To mitigate the over-smoothing effect in population-level generative models, we propose an encoder-guided latent inversion mechanism. A lightweight encoder performs fast initial mapping of fragmented observations, followed by adaptive refinement within the pre-trained latent space. This scheme captures unit-specific degradation characteristics and alleviates the regression-to-the-mean limitation of conventional models.
  2) Uncertainty-Aware Probabilistic RUL Inference. The repaired, degradation-consistent sequence is subsequently fed into the pre-trained Attention-BiLSTM network. Instead of performing a single deterministic forward pass, the network integrates an MCD module to execute stochastic forward sampling. This final step transforms deep temporal feature representations into uncertainty-aware RUL estimates, while providing an empirical estimate of the predictive epistemic uncertainty conditional on the reconstructed input (cf. Section 4.4 for what these intervals do and do not capture).

### 3.2. Macro-Anchored Generative Architecture

To learn a faithful prior distribution of equipment degradation, we propose the MAP-GAN architecture. The central design principle is to fuse global-stage awareness with local temporal dynamics. To achieve this, the model is structured around a macro-anchored micro-window mechanism: it restricts generation to a localized, fixed-length manifold—a micro-window of length *L* with each window explicitly conditioned on a discrete anchor index *c* that denotes its macroscopic operational stage. The structure of the proposed network is shown in Figure 2.

(1) Discrete Anchor Index Embedding Module

To represent both discrete degradation-stage semantics and continuous lifecycle positions within the latent space, we develop a dedicated cycle embedding module. Its core purpose is to project discrete equipment aging anchor points c∈N into unified semantic vectors with stage-perception capabilities, which can provide health-state guidance for both the subsequent generator and discriminator networks. To extract the semantic and temporal attributes of index anchors, we construct two mutually complementary feature representations: the first is a fine-grained semantic embedding used to distinguish different degradation stages, and the second is a time-sensitive encoding that describes the relative position of each stage within the whole equipment life cycle.

The fine-grained cycle embedding is obtained through a lookup table to extract discrete stage semantics, formulated as:

(6) $$v_{c}^{\mathrm{fine}}=E_{\mathrm{emb}}\left[c\right],\;v_{c}^{\mathrm{fine}}\in R^{d_{\mathrm{emb}}}$$

where $E_{\mathrm{emb}}$ refers to the embedding lookup table, and $d_{\mathrm{emb}}$ denotes the dimensionality of the embedding features. For the time-sensitive encoding, we scale the cycle anchor *c* into the interval [0,1] and mark the normalized result as $c_{\mathrm{norm}}$, so as to reflect its continuous relative position in the degradation lifecycle, followed by tanh activation and layer normalization to stabilize the feature distribution, given by:

(7) $$v_{c}^{\mathrm{time}}=\mathrm{LayerNorm}\left(\tanh(W_{t}\cdot c_{\mathrm{norm}}+b_{t})\right),\;v_{c}^{\mathrm{time}}\in R^{d_{\mathrm{emb}}}$$

where $W_{t}$ and $b_{t}$ are learnable network parameters.

To fuse the two complementary representations into one integrated stage-aware anchor vector, we first concatenate the two feature branches (marked as ⊕). The concatenated features are then fed into a linear projection layer, followed by LeakyReLU activation and layer normalization. The final output is expressed as:

(8) $$v_{c}=\mathrm{LayerNorm}\left(\mathrm{LeakyReLU}(W_{f}\left(v_{c}^{\mathrm{fine}}⊕v_{c}^{\mathrm{time}}\right)+b_{f})\right),\;v_{c}\in R^{d_{\mathrm{model}}}$$

where $W_{f}$ and $b_{f}$ denote additional trainable parameters, and $d_{\mathrm{model}}$ represents the latent dimensionality of the overall model. Compared with conventional lookup-based embedding methods that only capture discrete stage semantics, the proposed module combines discrete semantic features with continuous temporal position information, helping to quantify the relative lifecycle location of equipment degradation stages.

(2) Stage-Conditioned Generator ($G_{\psi }$)

Driven by the stage-aware anchor vector $v_{c}$ output from the cycle embedding module, we construct a stage-conditioned generator $G_{\psi }$. Because the latent input is unconstrained while battery capacity occupies a bounded operating range, the generator explicitly maps its output to an admissible capacity interval; in addition, it models the temporal correlation of degradation evolution and keeps the synthesized trajectories consistent with empirically observed degradation regularities. Its central role is to map raw Gaussian noise into degradation sequences that better conform to physically plausible degradation behavior.

The overall generation pipeline is implemented in four progressive steps. First, we adopt a hierarchical encoder to project the input Gaussian noise z∼N(0,I) into a high-dimensional latent space, which enhances the representation capability of noise features. The calculation is defined as:

(9) $$z_{\mathrm{feat}}=\mathrm{HierarchicalEncoder}\left(z\right),\;z_{\mathrm{feat}}\in R^{d_{\mathrm{model}}}$$

where $z_{\mathrm{feat}}$ denotes the encoded noise feature with a dimension of $d_{\mathrm{model}}$. Second, to capture the intrinsic temporal correlation of degradation evolution, we extend the noise feature along the time axis to match the micro-window length *L*. Subsequently, we further concatenate the expanded feature with the positional encoding PE to retain chronological information, and feed the fused sequence into stacked LSTM layers for temporal dynamics modeling:

(10) $$H_{G}=\mathrm{LSTM}\left(1z_{\mathrm{feat}}^{⊤}⊕\mathrm{PE}\right),\;H_{G}\in R^{L\times d_{\mathrm{model}}}$$

Third, to encourage consistency between the generated trajectory and the target degradation stage, we propose an asymmetric multi-head cross-attention mechanism as the generator’s core component. In this mechanism, the stage-aware anchor vector $v_{c}$ serves as the global query, which selectively focuses on the temporal features output by the LSTM—retaining fluctuations consistent with the target stage and filtering out patterns inconsistent with the expected stage. The attention calculation can be jointly formulated as:

(11) $$\left\{\begin{array}{c} Q=v_{c}\in R^{d_{\mathrm{model}}},\\ K=V=H_{G},\\ a_{c}=\mathrm{MultiHeadAttention}\left(Q,K,V\right)\in R^{d_{\mathrm{model}}} \end{array}\right.$$

where Q is the query vector, K and V are the key and value matrices, and $a_{c}$ denotes the anchor-conditioned context vector aggregated over the *L* temporal positions. Finally, to map the attention-refined feature into the valid physical range of degradation data, we integrate the attended feature $a_{c}$ with the stage anchor $v_{c}$. After fusing these two features, we feed the combined result into a linear projection layer, apply tanh activation to normalize the output, and subsequently scale the activated feature to match the physical interval $\left[x_{\min},x_{\max}\right]$. Detailed computational formulations are given below:

(12) $$\left\{\begin{array}{c} {\hat{X}}_{\mathrm{raw}}=\tanh\left(W_{o}\left(a_{c}⊕v_{c}\right)+b_{o}\right),\;{\hat{X}}_{\mathrm{raw}}\in R^{L},\\ \hat{X}=G_{\psi }\left(z|c\right)=x_{\min}+\left({\hat{X}}_{\mathrm{raw}}+1\right)\cdot \frac{x_{\max}-x_{\min}}{2},\;\hat{X}\in R^{L}. \end{array}\right.$$

Specifically, $W_{o}$ and $b_{o}$ are learnable network parameters, and $x_{\min}$ and $x_{\max}$ correspond to the lower and upper limits of the valid physical range for degradation data. Here, ${\hat{X}}_{\mathrm{raw}}\in R^{L}$ is the normalized intermediate window produced by the tanh activation (bounded within [−1,1]), and $\hat{X}\in R^{L}$ is the final stage-conditioned micro-window obtained after rescaling ${\hat{X}}_{\mathrm{raw}}$ into the physical interval $\left[x_{\min},x_{\max}\right]$. It should be noted that $\hat{X}$ operates at the micro-window granularity: during offline training, each full historical sequence $X_{i}^{\mathrm{hist}}\in R^{T_{i}}$ is partitioned into overlapping micro-windows of fixed length *L*, whereas the full-length reconstructed sequence ${\hat{X}}_{j}\in R^{T_{j}}$ of an online unit *j* (Section 2) is assembled by stitching the corresponding window-level outputs. Since the anchor embedding acts as a single global query, the attention output $a_{c}$ is a stage-aware context vector rather than a per-position sequence; the temporal profile of the generated window is produced by the output projection $W_{o}$ in Equation (12). Compared with conventional self-attention mechanisms that lack stage-oriented guidance, the asymmetric cross-attention mechanism leverages the stage vector as a global constraint, encouraging the physical consistency of the generated degradation trajectories.

(3) Joint-Authenticity Discriminator ($D_{\phi }$)

A discriminator that receives only the trajectory has no explicit mechanism for enforcing consistency between the generated sequence and its lifecycle stage. In light of this issue, we develop a joint-authenticity discriminator $D_{\phi }$ to assess the authenticity of trajectories more comprehensively. Concretely, the discriminator determines whether a trajectory satisfies two criteria: temporal realism and physical consistency with the stage anchor *c*.

To avoid redundant design, the proposed discriminator adopts a simple structure that shares the cycle embedding mechanism with the generator. It operates in three steps. We first utilize an LSTM to encode the input trajectory $X\in R^{L}$—a real micro-window sampled from $X_{i}^{\mathrm{hist}}$ or a synthesized one $\hat{X}=G_{\psi }\left(z|c\right)$ (cf. Equation (12))—and extract global temporal features, which model the inherent dynamic patterns of the trajectory. Subsequently, global average pooling is conducted on the LSTM output sequence to obtain a compact feature representation. The computation is defined as:

(13) $$H_{D}=\mathrm{LSTM}\left(X\right),\;s=\frac{1}{L}\sum_{t=1}^{L}h_{D,t},\;s\in R^{d_{\mathrm{model}}}$$

where $H_{D}$ is the output sequence of the LSTM, $h_{D,t}$ is the hidden state at time step *t*, and s denotes the compact temporal signature with a dimension of $d_{\mathrm{model}}$.

Second, we concatenate the temporal signature s with the cycle embedding vector $v_{c}^{\left(D\right)}$ to fuse the trajectory temporal characteristics and stage information. The vector $v_{c}^{\left(D\right)}$ follows the identical encoding paradigm as the cycle embedding module introduced in Section 3.2, helping to keep the representation of stage information consistent. The fused joint feature is fed into a linear layer with LeakyReLU activation to boost feature representation capability, which is formulated as:

(14) $$s_{\mathrm{joint}}=\mathrm{LeakyReLU}\left(W_{D1}\left(s⊕v_{c}^{\left(D\right)}\right)+b_{D1}\right),\;s_{\mathrm{joint}}\in R^{d_{\mathrm{model}}}$$

where $W_{D1}$ and $b_{D1}$ represent trainable parameters, and $v_{c}^{\left(D\right)}$ denotes the cycle embedding vector reused from the cycle embedding module. Finally, the joint feature $s_{\mathrm{joint}}$ is passed through a linear layer and a sigmoid activation function to predict the authenticity score ranging from 0 to 1 (1 denotes real trajectories and 0 denotes generated trajectories). This procedure is formulated as:

(15) $$D_{\phi }\left(X|c\right)=\sigma \left(W_{D2}s_{\mathrm{joint}}+b_{D2}\right),\;D_{\phi }\left(X|c\right)\in \left[0,1\right]$$

where σ is the sigmoid activation function, and $W_{D2}$ and $b_{D2}$ are trainable network parameters. By explicitly integrating temporal features and degradation stage information, the proposed discriminator helps reduce the limitation of conventional GAN discriminators, which may misclassify temporally smooth yet physically inconsistent trajectories as authentic.

We optimize the MAP-GAN framework via a conditional adversarial minimax game. Given the complete historical dataset $D_{\mathrm{hist}}$, the training objective is to minimize the Jensen–Shannon divergence between the synthetic distribution $P_{G_{\psi }}$ and the real data distribution $P_{\mathrm{data}}$, conditioned on the macro-anchor index *c*:

(16) $$\min_{\psi }\max_{\phi }V\left(D_{\phi },G_{\psi }\right)=E_{X∼P_{\mathrm{data}}\left(X|c\right)}\;\left[\log D_{\phi }\left(X|c\right)\right]+E_{z∼P_{z}\left(z\right)}\;\left[\log\;\left(1-D_{\phi }\left(G_{\psi }\left(z|c\right)|c\right)\right)\right]$$

By performing adversarial optimization within independent micro-windows, the proposed framework alleviates zero-padding artifacts in the learned generative manifold. This yields a degradation-consistent prior distribution, laying the foundation for the subsequent online sequence repair stage.

### 3.3. Physics-Informed Degradation Constraints

Many conventional generative frameworks depend entirely on Euclidean distance and adversarial loss for training. While such methods can yield mathematically smooth outputs, the resulting sequences may be inconsistent with the underlying physical properties of the equipment. For example, these models may violate degradation monotonicity or fail to capture sudden degradation knee-points. To better capture the intrinsic degradation regularities of equipment, we define the total objective function as:

(17) $$L_{\mathrm{total}}=\lambda_{\mathrm{adv}}L_{\mathrm{adv}}+\lambda_{\mathrm{hyb}}L_{\mathrm{hybrid}}$$

where $L_{\mathrm{adv}}$ denotes the generator-side adversarial term of the game defined in Equation (16), for which the non-saturating form $L_{\mathrm{adv}}=-E_{z∼P_{z}}\;\left[\log D_{\phi }\left(G_{\psi }\left(z|c\right)|c\right)\right]$ is adopted, and $L_{\mathrm{hybrid}}$ represents the proposed degradation-informed hybrid loss function. It jointly regularizes the absolute performance magnitude, stage-specific degradation trends, and micro-fluctuation dynamics:

(18) $$L_{\mathrm{hybrid}}=\lambda_{\mathrm{mag}}L_{\mathrm{mag}}+\lambda_{\mathrm{shape}}L_{\mathrm{shape}}+\lambda_{\mathrm{var}}L_{\mathrm{var}}$$

where $\lambda_{\mathrm{adv}}$, $\lambda_{\mathrm{hyb}}$, $\lambda_{\mathrm{mag}}$, $\lambda_{\mathrm{shape}}$, and $\lambda_{\mathrm{var}}$ are hyperparameters that balance the corresponding penalty weights; their values and selection strategy are summarized at the end of this subsection.

(1) Performance Amplitude Constraint ($L_{\mathrm{mag}}$)

The amplitude constraint regularizes the absolute numerical distribution of the generated sequence. For industrial equipment, this encourages the performance trajectory to comply with inherent operational boundaries and physical degradation limitations, helping to reduce non-physical numerical drift and unreasonable index jumps. In physical terms, $L_{\mathrm{mag}}$ anchors the absolute capacity level of the reconstructed trajectory to the plausible operational range of the equipment. We measure such discrepancy via the pointwise mean squared error (MSE) between the generated and real sequences:

(19) $$L_{\mathrm{mag}}=\frac{1}{BL}\sum_{b=1}^{B}\sum_{t=1}^{L}{\left({\hat{x}}_{b,t}-x_{b,t}\right)}^{2}$$

where ${\hat{x}}_{b,t}$ and $x_{b,t}$ denote the generated and ground-truth values, respectively, at cycle *t* of the *b*-th sample in a mini-batch; *B* is the mini-batch size; and *L* is the micro-window length. $L_{\mathrm{mag}}$ thus quantifies the pointwise amplitude discrepancy of the synthesized trajectory; step-wise changes are deliberately excluded here and are separately regularized by $L_{\mathrm{shape}}$, which keeps the functional boundary between the two terms unambiguous.

(2) Multi-order Degradation Shape Constraint ($L_{\mathrm{shape}}$)

The degradation of industrial equipment consistently exhibits distinct phase characteristics. It typically begins with a steady performance decline under normal operating conditions. Subsequently, accelerated degradation and obvious knee-points occur with long-term service and cumulative aging. To capture these universal evolutionary patterns, the proposed shape loss aligns the multi-order differential features of the sequences.

We adopt the forward-difference convention consistently throughout the paper. The first-order shape residual is defined as:

(20) $$\begin{array}{cc} r_{b,t}^{\left(1\right)} & =\Delta {\hat{x}}_{b,t}-\Delta x_{b,t}, \end{array}$$

(21) $$\begin{array}{cc} \Delta {\hat{x}}_{b,t} & ={\hat{x}}_{b,t+1}-{\hat{x}}_{b,t}, \end{array}$$

(22) $$\begin{array}{cc} \Delta x_{b,t} & =x_{b,t+1}-x_{b,t} \end{array}$$

The first-order residual $r_{b,t}^{\left(1\right)}$ constrains the fade-rate mismatch, while its temporal change captures the mismatch in degradation curvature. To maintain numerical stability against anomalous capacity spikes caused by sensor noise, we introduce the physical boundary projection operator $P_{\varepsilon_{1}}\left(\cdot \right)$ to map each first-order difference into a physically feasible range. The stabilized first-order residual is

(23) $${\tilde{r}}_{b,t}^{\left(1\right)}=P_{\varepsilon_{1}}\;\left(\Delta {\hat{x}}_{b,t}\right)-P_{\varepsilon_{1}}\;\left(\Delta x_{b,t}\right)$$

where the projection operator $P_{\varepsilon_{1}}\left(z\right)$ bounds the variation within the maximum allowable physical threshold $\left[-\varepsilon_{1},\varepsilon_{1}\right]$. It is worth noting that the boundary $\varepsilon_{1}$ is not heuristically preset; rather, it is established based on the historical statistical extremes of the degradation dataset. The operator is defined as:

(24) $$P_{\varepsilon_{1}}\left(z\right)=\left\{\begin{array}{cc} \varepsilon_{1}, & \mathrm{if}\;z>\varepsilon_{1}\\ z, & \mathrm{if}\;|z|\leq \varepsilon_{1}\\ -\varepsilon_{1}, & \mathrm{if}\;z<-\varepsilon_{1} \end{array}\right.$$

The corresponding stabilized second-order residual is defined as ${\tilde{r}}_{b,t}^{\left(2\right)}={\tilde{r}}_{b,t+1}^{\left(1\right)}-{\tilde{r}}_{b,t}^{\left(1\right)}$. By substituting these stabilized residuals, the comprehensive shape loss is constructed as follows:

(25) $$L_{\mathrm{shape}}=\frac{1}{B(2L-3)}\sum_{b=1}^{B}\left[\sum_{t=1}^{L-1}{\left({\tilde{r}}_{b,t}^{\left(1\right)}\right)}^{2}+\sum_{t=1}^{L-2}{\left({\tilde{r}}_{b,t}^{\left(2\right)}\right)}^{2}\right]$$

This projection strategy can alleviate gradient explosions while retaining the valid degradation trends.

(3) Fluctuation Variance Constraint ($L_{\mathrm{var}}$)

Real-world equipment degradation trajectories are rarely monotonically smooth. Instead, they contain high-frequency local dynamics induced by complex working conditions, environmental interference, and measurement noise. To discourage the generator from collapsing into an idealized, over-smoothed trend, we introduce a variance regularization term:

(26) $$L_{\mathrm{var}}=\frac{1}{B}\sum_{b=1}^{B}{\left[{\mathrm{Var}}_{t}\;\left(\Delta {\hat{x}}_{b,t}\right)-{\mathrm{Var}}_{t}\;\left(\Delta x_{b,t}\right)\right]}^{2}$$

where ${\mathrm{Var}}_{t}\left(\Delta x_{b,t}\right)$ denotes the temporal variance of the first-order differences in the real sequence. This constraint encourages the generated sequences to retain the stochastic fluctuation characteristics of the original data—i.e., to reproduce the genuine cycle-to-cycle fluctuation level of the monitored equipment rather than an idealized smooth trend. This term serves as a reconstruction regularizer rather than as an explicit estimator of aleatoric uncertainty.

In this study, the hyperparameter settings were determined based on preliminary experiments and fixed for all experiments. The adversarial weight is set to $\lambda_{\mathrm{adv}}=1$, and the hybrid physics loss is weighted by $\lambda_{\mathrm{hyb}}=0.5$. Within the hybrid loss, $\lambda_{\mathrm{mag}}=0.8$ is fixed, while the shape and variance weights follow a linear annealing schedule that increases from $\left(\lambda_{\mathrm{shape}},\lambda_{\mathrm{var}}\right)=\left(0.3,0.1\right)$ at 40% of the training horizon to (1.2,0.5) at 80%, so that shape-consistency requirements are progressively enforced after the generator achieves basic amplitude calibration. The projection bound $\varepsilon_{1}$ is derived from the statistical extremes of the first-order differences in the training set rather than being manually tuned. These hyperparameters are determined a priori after a small-scale pilot study and remain identical across all folds and datasets. Their necessity and robustness are examined in Section 4.7 through term-wise ablation experiments (w/o $L_{\mathrm{mag}}$, w/o $L_{\mathrm{shape}}$, w/o $L_{\mathrm{var}}$, and w/o all physics terms) and a physics-weight sensitivity analysis ($\lambda_{\mathrm{hyb}}\in \left\{0,0.25,0.5,1.0\right\}$).

### 3.4. Adaptive Latent Space Inversion for Instance-Level Repair

While the offline-trained MAP-GAN establishes a generalized, degradation-coherent manifold, directly sampling from this prior distribution yields a “population-averaged” trajectory. In practice, the deterioration behaviors of individual units are highly heterogeneous. To repair online block missingness while preserving instance-specific signatures, we propose an encoder-guided adaptive latent space inversion mechanism.

This online repair phase projects the fragmented observations back into the frozen MAP-GAN’s latent space to identify an instance-specific latent code $z_{j,c}^{⋆}$ that closely aligns with the degradation profile of online unit *j* at macro-anchor *c*.

(1) Lightweight Inversion Encoder ($E_{\omega }$)

To maintain notational consistency with Section 2, let $X_{j,c}^{\mathrm{obs}}\in R^{L}$ and $M_{j,c}\in {\left\{0,1\right\}}^{L}$ denote the length-*L* observed micro-window and its binary mask for online unit *j*, aligned with macro-anchor *c*. Each local reconstruction step ${\hat{X}}_{j,c}\in R^{L}$ contributes to the assembled full-length reconstruction output ${\hat{X}}_{j}\in R^{T_{j}}$.

For an online degraded sequence experiencing contiguous sensor failure, the inversion encoder $E_{\omega }$ takes the channel-wise concatenation of $X_{j,c}^{\mathrm{obs}}$ and $M_{j,c}$ as input. Through lightweight feature encoding, it directly maps the fragmented evidence into an initial instance-specific latent vector within the pre-learned latent manifold.

The encoder outputs a deterministic initial latent code as the optimization starting point:

(27) $$z_{j,c}^{\left(0\right)}=E_{\omega }\;\left(X_{j,c}^{\mathrm{obs}}⊕M_{j,c}\right)$$

where $z_{j,c}^{\left(0\right)}$ provides a high-quality initialization that inherits global degradation priors, accelerating subsequent latent-space optimization.

(2) Masked Physics-Informed Gradient Refinement

After extracting the initial latent vector $z_{j,c}^{\left(0\right)}$, we pair it with the macro-anchor index *c* and input the combination into the frozen pre-trained generator $G_{\psi }^{\mathrm{frozen}}$. The resulting initial local reconstruction is

(28) $${\hat{X}}_{j,c}^{\left(0\right)}=G_{\psi }^{\mathrm{frozen}}\;\left(z_{j,c}^{\left(0\right)}|c\right)$$

To encourage the inverted trajectory to align with the specific context of each individual online unit, we move away from traditional point-wise reconstruction methods. Instead, we employ a Masked Hybrid Physical Loss which incorporates masking logic into the previously presented $L_{\mathrm{hybrid}}$. Specifically, the inversion loss is defined over the observable regions. This refinement process encourages the generator to match the visible portions of the sequence, while the GAN’s inherent prior is allowed to fill in the missing gaps.

(29) $$\begin{array}{cc} L_{\mathrm{inv}}\left(z\right)\; & =L_{\mathrm{hybrid}}\;\left(G_{\psi }^{\mathrm{frozen}}\left(z|c\right),X_{j,c}^{\mathrm{obs}};M_{j,c}\right),\\ z_{j,c}^{⋆}\; & =\arg\min_{z}L_{\mathrm{inv}}\left(z\right). \end{array}$$

The semicolon before $M_{j,c}$ indicates that the hybrid loss is evaluated only on observable entries specified by the mask. For the first- and second-order terms, only differences whose required consecutive samples are all observed are included; differences crossing a missing-block boundary are excluded.

Starting from $z_{j,c}^{\left(0\right)}$, iterative gradient refinement yields the instance-customized code $z_{j,c}^{⋆}$. The optimized local repair is ${\hat{X}}_{j,c}=G_{\psi }^{\mathrm{frozen}}\left(z_{j,c}^{⋆}|c\right)$, and the collection of local repairs is assembled into the full reconstructed sequence ${\hat{X}}_{j}$. This procedure preserves degradation-consistent, instance-specific dynamics while alleviating the “regression-to-the-mean” limitation.

### 3.5. Uncertainty-Aware RUL Prediction via Attention-BiLSTM-MCD

To quantify the predictive uncertainty associated with the reconstructed sequences (Section 3.4), this framework introduces an uncertainty-aware prediction module. Grounded in the physical degradation laws and temporal coherence of reconstructed CM sequences, the module integrates deterministic feature extraction and probabilistic inference to yield robust RUL estimations. In quantifying predictive uncertainty, this framework distinguishes between aleatoric and epistemic uncertainties. $L_{\mathrm{var}}$ preserves the empirical level of cycle-to-cycle fluctuations in the reconstructed sequence; it is not used as an explicit estimator of aleatoric uncertainty. The present uncertainty analysis therefore focuses on predictor-side epistemic uncertainty conditional on the reconstructed input, which arises from model parameterization and incomplete information under block missingness. By activating MCD during inference, the Attention-BiLSTM performs an approximate Bayesian variational estimate of the predictive epistemic uncertainty of the predictor, conditional on the reconstructed input; the intervals therefore characterize the predictor-side uncertainty given a reconstruction, and their relation to the upstream reconstruction quality is examined empirically in Section 4.4.

(1) Deterministic Feature Extraction (Attention-BiLSTM)

The core feature extractor adopts a BiLSTM network augmented with an attention mechanism, which is designed to capture both long-range temporal dependencies and critical degradation turning points from reconstructed CM sequences that retain physical plausibility and temporal consistency.

A sliding window strategy first converts univariate degradation sequences into fixed-length temporal segments, which serve as input to the BiLSTM encoder. The encoder computes hidden states in both forward and backward directions simultaneously, and captures complete temporal context by concatenating the two directional outputs at each time step. This bidirectional encoding scheme leverages the continuous degradation patterns recovered from block-missing CM data via temporal reconstruction, alleviating information fragmentation caused by persistent data voids.

To mitigate information dilution across long sequences and emphasize informative degradation phases, a learnable attention layer assigns dynamic weights to each temporal step. A scalar attention score is first computed for each BiLSTM hidden state and then normalized across the full window:

(30) $$e_{t}=w_{a}^{⊤}h_{t}+b_{a},\;\alpha_{t}=\frac{\exp(e_{t})}{\sum_{\tau =1}^{W}\exp\left(e_{\tau }\right)}$$

(31) $$h_{\mathrm{att}}=\sum_{t=1}^{W}\alpha_{t}\;h_{t},\;\sum_{t=1}^{W}\alpha_{t}=1$$

where $w_{a}$ and $b_{a}$ are trainable attention parameters, $h_{t}\in R^{2d_{h}}$ denotes the concatenated forward and backward BiLSTM hidden state at cycle *t* ($d_{h}$ is the per-direction hidden dimensionality), $\alpha_{t}$ is the normalized attention weight at cycle *t*, and *W* represents the length of the input temporal window. Building upon the aggregated feature, a hierarchical fully connected network maps the temporal representation to the RUL target. Dropout layers are embedded within the fully connected stack, acting both as a regularizer during training and as the architectural basis for subsequent probabilistic inference.

(2) Probabilistic Inference via Monte Carlo Dropout

While Dropout is conventionally employed exclusively during training for regularization, theoretical analysis suggests that retaining active Dropout during inference approximates Bayesian variational inference. This mechanism is used to approximate the predictor-side epistemic uncertainty of the RUL prediction conditional on a fixed reconstructed input, without additional architectural modifications.

During online inference, the model remains in stochastic mode with Dropout layers activated. For each reconstructed RUL input window ending at cycle *t* of online unit *j*, *S* independent forward passes are performed, with different subsets of neurons randomly masked in each pass (S=20 in the main setting; Table 1). This process effectively samples *S* predictor realizations from the approximate posterior distribution over network parameters, yielding the ensemble ${\left\{{\hat{y}}_{j,t}^{\left(s\right)}\right\}}_{s=1}^{S}$.

Two key operational metrics are derived from this empirical predictive distribution, both related to the quality of temporal reconstruction and the physical plausibility of CM sequences:

(1) RUL Point Estimate

The expected value of the prediction ensemble balances accuracy and robustness against sampling noise, providing a stable estimate aligned with the dominant physical degradation trend:

(32) $${\hat{\mu }}_{j,t}=\frac{1}{S}\sum_{s=1}^{S}{\hat{y}}_{j,t}^{\left(s\right)}$$

(2) Epistemic Uncertainty Quantification

The standard deviation of the predictions approximates the model’s confidence in its estimation, which reflects the fidelity of temporal reconstruction for block-missing data:

(33) $${\hat{\sigma }}_{j,t}=\sqrt{\frac{1}{S-1}\sum_{s=1}^{S}{\left({\hat{y}}_{j,t}^{\left(s\right)}-{\hat{\mu }}_{j,t}\right)}^{2}}$$

Assuming an approximately Gaussian distribution of predictions, a two-sided interval with a nominal coverage level of 1−β is constructed as $\left[{\hat{\mu }}_{j,t}-z_{1-\beta /2}{\hat{\sigma }}_{j,t},\;{\hat{\mu }}_{j,t}+z_{1-\beta /2}{\hat{\sigma }}_{j,t}\right]$; for a nominal 95% interval, β=0.05 and $z_{1-\beta /2}\approx 1.96$. This interval quantifies the predictive epistemic uncertainty of the predictor conditional on the reconstructed input. It should not be interpreted as a direct measure of upstream reconstruction quality: the calibration analysis in Section 4.4 shows that the association between the interval width and the reconstruction error is weak, whereas the reconstruction error itself propagates clearly into the point-estimate accuracy. As an illustrative post-hoc analysis, a width inflation of approximately 1.3× would approach the nominal 95% coverage on the present evaluation set.

## 4. Experimental Validation

### 4.1. Dataset and Setup

(1) Dataset Description and Preprocessing

We used CM data of 9 Lithium-ion button cells, acquired via a NEWARE CT-4008Tn-5V10mA-164 high-precision cell tester (Neware Technology Limited, Shenzhen, China), to evaluate the proposed method [40]. Each cycle included constant current (CC) charging and 0.5C constant current discharging. Consistent with industrial PHM standards and lithium-ion cell degradation test criteria, the end-of-life (EOL) threshold was defined as 30% capacity degradation—i.e., a cell fails when its discharge capacity drops to 70% of the initial rated capacity. To examine the method’s effectiveness and robustness, we adopted Leave-One-Out Cross-Validation (LOOCV): Each cell was held out once as the test cell, while the remaining eight cells were used to build the offline prior knowledge base for both MAP-GAN and the downstream Attention-BiLSTM. This rotation encourages the performance metrics to reflect generalization rather than overfitting.

Due to the limited availability of complete run-to-failure battery degradation datasets with continuous block missingness, this study focuses on validating the proposed framework under a controlled LOOCV setting. Larger-scale validation under diverse operating conditions will be investigated in future work.

To simulate severe real-world data loss, we designed a dual-window simulation pipeline whose masking protocol is fully specified as follows. Continuous block gaps of this kind are motivated by practical failure modes of monitoring systems: sustained communication-bus interruptions, sensor offline periods, periodic system sleep modes, and transient acquisition faults typically disable logging for many consecutive operating cycles rather than producing isolated point dropouts. The block-missing protocol therefore targets these structurally different gaps. In the main experiments, a 65-cycle sliding generative window (L=65, step size 10) scanned each cell’s lifecycle; within each window, a contiguous interior block of 39 cycles—indices [10,49)—was masked, corresponding to a 60% localized missing ratio with 10 leading and 16 trailing observed context cycles. The masked measured entries serve as the ground truth for evaluating reconstruction quality. This configuration is deliberately challenging: the gap is too long for local interpolation to bridge, yet sufficient bidirectional context remains for bounded-gap reconstruction (cf. Section 2.2). To examine robustness beyond this single setting, Section 4.5 additionally varies the missing ratio (20%, 40%, 60% at the interior position) and the missing position (early-position block starting at cycle 0 and late-position block ending at the window boundary, both of 60% ratio). All compared methods share exactly the same masking sequences, window partitioning, and evaluation segments, ensuring that inter-method differences stem only from the repair quality. For downstream RUL prediction, the sliding window was reduced to W=25 cycles to capture the relationship between capacity trends and RUL.

(2) Training and Evaluation Protocol

Experiments were conducted on a workstation with an NVIDIA GeForce RTX 3060 GPU, Intel Core i5-12450H CPU, 16 GB RAM, and Windows 11 (64-bit). The framework was implemented in Python 3.8 with PyTorch. Hyperparameter configurations for MAP-GAN and Attention-BiLSTM-MCD are summarized in Table 1.

MAP-GAN is trained with joint adversarial optimization from the first epoch: the generator and the discriminator are updated alternately (one generator update per discriminator update), and the generator gradients are clipped at a maximum norm of 1.0. The generator is optimized using Adam with a learning rate of $1\times 10^{-4}$, $\beta_{1}=0.5$, $\beta_{2}=0.9$, and weight decay $1\times 10^{-5}$; cosine annealing is applied over the full training horizon. The discriminator is optimized using Adam with a learning rate of $1\times 10^{-4}$, $\beta_{1}=0.5$, and $\beta_{2}=0.9$, and uses no weight decay. The adversarial weight is fixed at $\lambda_{\mathrm{adv}}=1$ and the hybrid physics-loss weight at $\lambda_{\mathrm{hyb}}=0.5$, consistent with Section 3.3; only the internal shape/variance weights of $L_{\mathrm{hybrid}}$ follow the annealing schedule described there.

We pre-train the prognostic network on the full historical dataset with MSE loss and Adam optimizer (weight decay $1\times 10^{-5}$). Training runs for 100 epochs at batch size 32, with an initial learning rate of $1\times 10^{-3}$ that halves every 20 epochs. The checkpoint with the lowest validation MAE is retained for inference.

(3) Baseline implementation and tuning protocol

All deep imputation baselines (SAITS, BRITS, CSDI, and GP-VAE) are implemented via the PyPOTS library and trained with a unified budget of at most 200 epochs with early-stopping patience of 20 epochs, where the validation set is the last training cell of each fold (never the test cell), and the checkpoint with the lowest validation MSE is retained. For BRITS, which performs atypically, the training budget was extended from the initial 30 epochs to the full 200-epoch budget and the RNN hidden size and learning rate were examined within their recommended ranges; the best-validation epoch appears early (below 30 in most folds) and prolonged training does not improve the validation MSE, so the poor test performance under 60% contiguous gaps is unlikely to be explained solely by an insufficient training budget. We therefore report these results as an observation under the evaluated setting rather than as evidence of a deficiency of the BRITS method family itself. WGAN-GP follows the same masking and evaluation protocol with the gradient penalty coefficient fixed at 10 and the learning rate evaluated at $\left\{10^{-4},5\times 10^{-5}\right\}$. All baselines share exactly the same masks, windows, and downstream predictor as MAP-GAN. All learnable methods are trained on the training-side cells of the corresponding fold and never access the test cell; the deep imputation baselines reserve the last training cell for validation-based early stopping, following common practice for these method families.

(4) Reproducibility

Our experiments are implemented with PyTorch 2.7.1+cu128 and NumPy 2.2.6, and all evaluations use seed 44; reported results are single-seed, and the seeds and trained checkpoints are released for reproduction. The complete configuration includes: 500 GAN training epochs per fold with joint adversarial updates from the first epoch; generator Adam ($1\times 10^{-4}$, cosine annealing, gradient clipping at norm 1.0); discriminator Adam ($1\times 10^{-4}$); the RUL predictor trained for 100 epochs with the best-validation-MAE checkpoint; deep baselines trained for at most 200 epochs with patience 20 and best-validation-MSE checkpointing. The run-to-run variability quoted below was estimated from auxiliary evaluation runs that used different random seeds and is approximately 0.5–1 cycle in RUL MAE.

(5) Computational cost

Table 2 reports the model complexity and the measured per-input-window computation times. All runtime benchmarks in Table 2 were independently re-measured on a single NVIDIA GeForce RTX 4080 SUPER GPU; these timings are intended for relative computational comparison and are separate from the workstation used for the main experiments. The reconstruction/imputation rows correspond to one 65-cycle window (batch size one), and the MCD row corresponds to 20 stochastic passes over one 25-cycle RUL window. MAP-GAN’s per-window reconstruction cost (2.43 s for 200 latent-inversion steps) is higher than the forward-only imputation of the deep baselines, because latent inversion performs iterative optimization rather than a single forward pass; this is the trade-off for instance-specific reconstruction. All other components are lightweight (the Attention-BiLSTM-MCD inference costs about 1.1 ms per window for the full 20-sample ensemble).

We evaluate imputation quality from two angles: statistical match to ground truth and fidelity to physical degradation patterns. All metrics are computed only on the masked index set $\Omega^{\mathrm{miss}}$ (the online-unit subscript is omitted in this metric definition for brevity), with $N_{\mathrm{miss}}=\left|\Omega^{\mathrm{miss}}\right|$. Table 3 lists the metrics used: Mean Absolute Error (MAE), Root Mean Squared Error (RMSE), Mean Absolute Percentage Error (MAPE), Pearson correlation coefficient (*r*), and Kolmogorov–Smirnov (KS) statistic for statistical similarity; Negative Difference Ratio (NDR), Degradation Rate Coefficient of Variation (DegRate_CV), residual standard deviation, volatility, and Sign Change Rate (SCR) for degradation and temporal fidelity. The repaired and held-out measured capacities are denoted by ${\hat{x}}_{t}$ and $x_{t}$, respectively.

Here, NDR, CV, and SCR denote the negative-difference ratio, the coefficient of variation of the first-order differences, and the sign-change ratio of the first-order differences, respectively. With the forward-difference convention $\Delta x_{t}=x_{t+1}-x_{t}$, the negative-difference ratio is $\mathrm{NDR}\left(X\right)=\frac{1}{N_{\mathrm{miss}}-1}\sum_{t:\;t,t+1\in \Omega^{\mathrm{miss}}}I\left(\Delta x_{t}<0\right)$ for a contiguous masked segment. Following the principle that a faithful reconstruction should reproduce the true dynamic behavior rather than merely behave “smoothly”, the degradation and temporal-fidelity metrics are defined as the absolute deviations between the corresponding dynamic characteristics of the reconstructed and ground-truth sequences; a lower deviation therefore indicates better consistency with the actual degradation dynamics.

We assess prediction quality on two fronts: point-estimate accuracy and uncertainty calibration. Table 4 summarizes the metrics. For accuracy, we use MAE, RMSE, SMAPE, and $R^{2}$. For uncertainty, we report coverage probability (CP), interval width (PI_Width), and the Coverage Width-based Criterion (CWC), which penalizes under-coverage while rewarding tighter intervals. In these aggregate metric definitions, $n=1,\ldots ,N_{\mathrm{eval}}$ indexes evaluated prediction points, thereby avoiding reuse of the lifecycle index *t*. The interval bounds are denoted by ${\hat{y}}_{n}^{L}$ and ${\hat{y}}_{n}^{U}$. CWC uses target coverage γ=0.95 and penalty η=50. Normalized Mean Prediction Interval Width (NMPIW) normalizes PI_Width by the observed RUL range. Specifically, $\mathrm{NMPIW}=\mathrm{PI}\_\mathrm{Width}/(y_{\max}-y_{\min})$.

### 4.2. Effectiveness of Missing Data Imputation

To systematically examine the efficacy of the generative imputation phase, we benchmark the proposed MAP-GAN against both traditional statistical/interpolation methods and generative models. Specifically, the proposed method is compared with Mean Fill, Spline Interpolation, SAITS [37] and BRITS [36] from the PyPOTS [41] library, and a TCN-based WGAN-GP [42] model. All evaluations follow the LOOCV protocol and use the metrics defined above, covering two complementary dimensions: statistical similarity and degradation-temporal fidelity. Notably, the degradation-temporal metrics are computed as the absolute differences between the imputed sequence and the ground-truth sequence in terms of their corresponding dynamic characteristics, where lower values indicate better consistency with the true degradation behavior. The results of statistical similarity and degradation-temporal fidelity among these methods are presented in Figure 3.

For statistical similarity (Figure 3a), we perform a quantitative comparison between MAP-GAN and all baseline models. Averaged over the nine test cells, MAP-GAN attains the lowest pointwise errors (MAE 2.26 and RMSE 3.09), the lowest MAPE (2.20%), and the smallest KS statistic (0.250), while its Pearson correlation coefficient (*r*) (0.858) is comparable to that of SAITS (0.861), indicating its ability to retain the underlying statistical distribution of the raw data. In contrast, BRITS and WGAN-GP exhibit noticeable over-smoothing and mode-collapse-like behavior, while Mean Fill and Spline Interpolation show weak distributional agreement with the ground truth.

For degradation characterization and temporal fidelity (Figure 3b), all metrics are evaluated as absolute deviations from the ground-truth dynamic characteristics, so a lower value indicates a closer reproduction of the true degradation behavior rather than merely a smoother output. On these deviation-based metrics, MAP-GAN and SAITS are the two most faithful methods, each leading on complementary characteristics: MAP-GAN achieves the smallest sign-change deviation (12.8 vs. 24.4 percentage points for SAITS), indicating that the trend-reversal rhythm of its reconstruction best matches the true fluctuation pattern, whereas SAITS attains smaller volatility and degradation-rate-CV deviations, with MAP-GAN following closely. The remaining baselines fail in characteristic ways: the flat fill of Mean Fill contradicts the monotone fade (largest NDR deviation), WGAN-GP inflates the volatility deviation by an order of magnitude, and BRITS distorts both the trend and the fluctuation level. These results indicate that MAP-GAN suppresses non-physical capacity recoveries, keeps the degradation rate broadly consistent with the actual aging process, and preserves fine-grained temporal fluctuations, while no single method dominates every dynamic characteristic.

To examine the robustness of the imputation method across the full cell degradation lifecycle, four representative degradation segments are selected from Cell #1 for qualitative performance comparison across distinct aging phases: the early stable degradation phase (Seg. 1), mid-term steady degradation phase (Seg. 2), degradation knee-point transition phase (Seg. 3), and late accelerated aging phase (Seg. 4). As can be observed from Table 5, MAP-GAN delivers consistently competitive accuracy across all four segments (MAE 0.74–4.12) without any segment-level failure, and attains the best sign-change fidelity at the knee-point segment, where the fade-rate structure changes most markedly. Each baseline, in contrast, exhibits at least one weak segment: Spline Interpolation is the most accurate in the early segment but degrades noticeably from the knee-point onward, SAITS is strong in the mid and late segments yet is less accurate in the early segment, and Mean Fill, BRITS, and WGAN-GP are uncompetitive throughout. MAP-GAN is thus the only method whose accuracy remains stable across all lifecycle phases.

Figure 4 visually confirms the stage-wise differences reported in Table 5 for four representative missing segments. Mean Fill cannot reproduce the temporal evolution within the gap, while Spline Interpolation becomes increasingly biased around the knee-point and late-life segments. The deep baselines exhibit model-specific deviations, whereas MAP-GAN maintains the overall degradation slope together with the local fluctuations across the four displayed stages.

### 4.3. Results of RUL Prediction

To examine the prognostic performance of the imputed sequences, the complete sequences reconstructed by all comparison methods are fed into an identical, pre-trained Attention-BiLSTM-MCD predictor. Using the same downstream predictor for all methods ensures that differences in RUL performance primarily reflect the quality of upstream reconstruction rather than differences in predictor architecture. To further examine the generalization of the proposed model, we adopt the same LOOCV protocol as in the imputation phase and conduct prognostic experiments on all nine test cells. The comparison covers the proposed MAP-GAN, a classic conditional-GAN baseline (Vanilla CGAN with a standard BCE objective), the deep imputation baselines (SAITS, BRITS, CSDI, and GP-VAE), the traditional baselines (Mean Fill and Spline Interpolation), and the True-Cap reference that predicts directly from the intact capacity sequence; all methods are evaluated under the identical main-setting masks. WGAN-GP, which participates only in the imputation comparison of Section 4.2, is not included in this prognostic comparison. Table 6 summarizes the average prognostic performance across all test cells based on the metrics defined in Table 4: MAE, RMSE, and $R^{2}$ are reported as per-cell mean ± SD, whereas CP, PI_width, and CWC are computed over the pooled windows of all cells (CWC uses γ=0.95 and η=50). MAP-GAN attains an RUL MAE of 11.57±9.65 cycles (mean ± SD over the nine test cells), numerically comparable to the complete-data reference (11.40±9.86) and to the strongest baseline SAITS (11.46±10.35); because the downstream predictor itself carries approximation error, the complete-data input is an empirical reference rather than a strict numerical lower bound. The classic conditional-GAN baseline, trained with a purely adversarial objective, degrades noticeably (Vanilla CGAN 14.73±9.54), and the deep imputation baselines behave consistently with the imputation layer: SAITS remains competitive, whereas BRITS and GP-VAE are unreliable under 60% contiguous gaps and CSDI degrades severely. MAP-GAN also achieves the best $R^{2}$ among the repair methods and a coverage probability close to the reference.

Regarding statistical description, given the limited number of test cells (n=9), no formal significance claim is made; the cross-cell mean ± SD is reported instead. MAP-GAN, SAITS, and the complete-data reference exhibited similar mean errors relative to the observed cross-cell variability, whereas the mean errors of the remaining baselines are 2–6 cycles higher. We note that with only nine cells, formal paired significance testing would have limited power and would be sensitive to distributional assumptions; the robustness of the proposed method is therefore argued through the sensitivity analysis (Section 4.5), the external validation (Section 4.6), and the ablation studies, rather than through single-scenario significance claims.

To further illustrate the per-cell behavior of all methods, Figure 5 presents a heatmap of the per-cell RUL MAE for the nine methods (the eight repair methods plus the True-Cap reference) on the test cells (main setting; logarithmic color scale with the raw values annotated; the BRITS and GP-VAE rows exceed 200 cycles and are therefore compressed visually). No single method dominates every cell: MAP-GAN and SAITS attain the best average ranks (3.6 and 3.4 out of the eight repair methods), the cells on which MAP-GAN is weakest are also challenging for the other strong methods, and Vanilla CGAN attains the lowest single-cell errors only on Cells 1 and 3 at the cost of clearly worse cross-cell averages, whereas the failure of BRITS and GP-VAE is uniform across all cells.

Figure 6 presents the MAP-GAN RUL trajectories—the mean estimate together with the nominal 95% MCD interval obtained from 20 stochastic forward passes—on three representative test cells: the best case (Cell #8, MAE 3.71), the median case (Cell #7, MAE 7.67), and the most difficult case (Cell #6, MAE 29.23). On the best and median cells, the predicted trajectories closely follow the true RUL trend, and the interval width varies across the lifecycle and across cells. The difficult Cell #6 exhibits a systematic deviation together with substantial predictive dispersion, illustrating the larger cell-dependent prognostic difficulty observed in the cross-cell results.

The above results support the effectiveness of the proposed MAP-GAN framework for RUL prediction under continuous block missing data scenarios.

### 4.4. Uncertainty Calibration and Error Propagation

Beyond the 95% coverage statistics reported in Table 6, we further examine (i) how well the MCD-based predictive intervals are calibrated across nominal coverage levels, and (ii) how the upstream reconstruction error propagates into the downstream RUL error and uncertainty.

For MAP-GAN, the predictive intervals are constructed as ${\hat{\mu }}_{j,t}\pm z_{1-\beta /2}{\hat{\sigma }}_{j,t}$, where 1−β is the nominal coverage and $z_{1-\beta /2}$ is the corresponding standard-normal quantile, and the empirical coverage is measured over all missing-overlapping windows pooled across the nine test cells. The results are 42.7% (nominal 50%), 58.2% (nominal 80%), 69.5% (nominal 90%), and 76.7% (nominal 95%). As expected for nested Gaussian intervals, the empirical coverage increases with the nominal level; however, systematic under-coverage is observed at all levels, with the empirical coverage falling 7–22 percentage points below the nominal level, i.e., the MCD intervals are somewhat optimistic (too narrow) rather than over-conservative at the 95% level. As an illustrative post-hoc analysis, inflating the interval width by a factor of approximately 1.3 on the present evaluation set would approach the nominal 95% coverage; in practical deployment, the inflation factor should be estimated exclusively from an independent validation set.

Pairing the per-cell reconstruction errors with the per-cell RUL metrics over all methods and cells (9 methods × 9 cells, i.e., 81 pairs) yields a pooled descriptive Spearman association of ρ=0.76 between reconstruction RMSE and downstream RUL RMSE (Pearson r=0.50). Because observations from the same cell are not independent, the association is quantified with a cell-clustered bootstrap (2000 resamples; cells resampled with replacement; the methods are treated as fixed comparison conditions), giving a 95% confidence interval of [0.60,0.88], which supports a strong monotone propagation of upstream reconstruction quality into the prognosis. Restricting the analysis to the non-collapsed methods, the association remains positive (ρ=0.44, cluster-bootstrap 95% CI [0.07,0.70]), indicating that even within the competitive regime, better reconstruction tends to yield better prognosis. In contrast, the association between the reconstruction error and the predictive uncertainty (the mean predictive standard deviation) is weak (r=0.23; cluster-bootstrap 95% CI covering zero), suggesting that the MCD interval width mainly reflects the epistemic variability of the predictor itself rather than the upstream imputation error; uncertainty-aware fusion of the imputation posterior into the interval construction is therefore a meaningful direction for future work.

### 4.5. Sensitivity to Missing Patterns

The main experiments adopt a fixed 60% interior block-missing configuration. To examine how sensitive the downstream prognostic performance is to the missing pattern itself, we evaluate five scenarios on the same nine test cells: three interior-position missing ratios, R20% (13 masked cycles), R40% (26 masked cycles), and R60% (39 masked cycles, the main setting), plus two 60%-ratio position variants, P-early (the block starts at cycle 0 of the window, leaving only right-end context) and P-late (the block ends at the window boundary, leaving only left-end context). All methods, including the deep imputation baselines, are re-evaluated under exactly the same masks, and the reconstructed sequences are fed into the identical Attention-BiLSTM-MCD predictor. The True-Cap reference (prediction directly from the intact capacity sequence) serves as the complete-data reference for each scenario. Table 7 reports the mean RUL MAE over the nine test cells.

Table 7 shows that MAP-GAN is largely insensitive to the interior missing ratio: across R20%, R40%, and R60%, it stays within 0.2 cycles of the complete-data reference and is the only method whose gap to the reference does not widen as the interior gap grows from 20% to 60%. A clearer difference emerges when the missing block moves to a window boundary: MAP-GAN degrades only mildly, whereas all baselines deteriorate substantially—Spline Interpolation collapses by more than an order of magnitude because one-sided extrapolation breaks its interpolation premise, BRITS and GP-VAE remain unreliable, and even the strongest baseline SAITS roughly doubles its error at P-late. Consequently, MAP-GAN exhibits the smallest worst-case deviation from the reference (2.2 cycles) and is the only method that never exhibits a catastrophic failure under any evaluated pattern, indicating that its performance is not an artifact of a single favorable missing configuration.

### 4.6. External Validation on a Second Dataset

To further examine the cross-dataset generalization of the proposed framework beyond the primary nine-cell dataset, we additionally evaluate it on an independent, publicly available lithium-ion battery dataset collected by the Hawaii Natural Energy Institute (HNEI) (The HNEI dataset contains fourteen 18,650-format cells with graphite/NMC-LCO chemistry cycled under a unified profile; each cell provides more than 1000 valid discharge-capacity records) [43], hereafter referred to as the HNEI dataset. Since this validation serves as a supplementary external check rather than a full replication, the protocol is kept as close as possible to the primary experiments while following a random-split scheme suited to the larger cell count: three random train/test splits were generated; within each split, nine cells were assigned to training and the remaining cells to testing, and a given cell may appear in the training set of one split and the test set of another (cells are re-sampled across splits, and the splits themselves are not jointly exhaustive partitions). The first 650 cycles of each cell are used, the same 65-cycle window, 60% interior block-missing protocol, and the same five missing-pattern scenarios of Section 4.5 are applied, and the failure threshold is defined as 0.7× the cycle-4 capacity. MAP-GAN, the classic conditional-GAN baseline (Vanilla CGAN), the representative deep imputation methods (SAITS, BRITS, CSDI, GP-VAE), the traditional baselines, and the True-Cap reference are all retrained and re-evaluated on this dataset under identical budgets. Table 8 reports the RUL MAE as the mean ± SD over the three splits (each split value is the mean over its test cells).

The external validation reproduces the main findings of the primary dataset. MAP-GAN remains within approximately one cycle of the complete-data reference at the interior missing positions, attains the best early-position result, and its late-position degradation (+2.8 cycles over the reference) is comparable to that of the strongest baseline SAITS (+2.9) and clearly smaller than those of Vanilla CGAN (+5.0) and Spline Interpolation (+172.6). The failure modes observed on the primary dataset are likewise replicated: Spline Interpolation deteriorates sharply at one-sided missing positions, and BRITS, CSDI, and GP-VAE degrade severely under 60% contiguous gaps. MAP-GAN again exhibits the smallest worst-case deviation among the repair methods, indicating that the conclusions drawn from the primary nine-cell LOOCV study are not dataset-specific.

### 4.7. Model Mechanism Interpretation and Ablation Analysis

While the aforementioned experiments have examined the imputation and prognostic performance of the proposed MAP-GAN framework, this subsection examines the operating mechanism of MAP-GAN and the contribution of its main components. Specifically, the latent space structure and instance-level inversion trajectory are first visualized to qualitatively illustrate the operating principle of the model, and ablation experiments are subsequently conducted to quantitatively examine the contribution of each key module. Together, these analyses provide a controlled mechanism study of the MAP-GAN framework on the held-out cell.

(1) Latent space structure

Figure 7 visualizes the latent space distribution learned by the proposed MAP-GAN generator with macro-anchoring design. As shown in the PCA representation on the right, PC1 explains 93.0% of the latent variance, and PC2 explains an additional 4.8%, giving a cumulative explained variance of approximately 97.8% for the first two components, indicating that the temporal degradation trend of battery capacity can be largely characterized within a low-dimensional subspace. The sample points change gradually from blue (early aging cycles) to red (late aging cycles) in color distribution, which suggests that the macro-anchoring module embeds battery aging progression into latent representations. The observed stage-wise organization is consistent with the intended lifecycle conditioning introduced by macro-anchoring.

The t-SNE visualization on the left further reveals the latent space clustering characteristic. Samples belonging to different degradation stages form relatively independent clusters with discernible boundaries. No obvious mode collapse or feature confusion is observed across the feature space. These results indicate that the macro-anchored latent space is organized according to the aging pattern of the batteries. Such a structured latent representation provides a basis for missing sequence repair and latent inversion in the subsequent imputation stage.

(2) Inversion trajectory

Built upon the structured latent space established by macro-anchoring, Figure 8 presents the latent inversion optimization process of MAP-GAN for missing degradation sequence repair, from both global overview and local magnification perspectives. In the macro landscape on the left, the contour color indicates the reconstruction MSE, where warm-color regions correspond to low loss and better imputation performance. Most randomly sampled latent points scatter in high-loss areas, while the encoder-derived initial latent vector $z_{j,c}^{\left(0\right)}$, conditioned jointly with the macro-anchor *c* (cf. Section 3.4), lies relatively close to the optimized solution $z_{j,c}^{⋆}$ and falls in a low-loss region. This provides a favorable initial starting point and simplifies the subsequent optimization search.

The local enlarged view on the right displays the detailed optimization trajectory. In the displayed two-dimensional projection, the loss surface appears smooth, and the gradient descent path moves steadily toward the optimal point in an approximately straight line, with no severe oscillation or redundant iteration. The inversion process can converge to a satisfactory solution within only hundreds of steps. By leveraging the degradation pattern learned from battery data, MAP-GAN narrows the latent search range, reduces computational overhead, and supports efficient online missing data imputation.

(3) Ablation experiments

To quantitatively examine the independent contribution of each core module under controlled conditions, systematic ablation experiments are conducted with all training configurations and hyperparameters kept consistent except for the removed target module. All variants are evaluated on the held-out Cell #1 with ten masked windows under the same protocol as Section 4.5, so that the module ablation, the physics-term ablation in Table 9, and the sensitivity analysis share a common evaluation anchor. The quantitative results are summarized in Table 10.

Two observations arise from Table 10. First, removing the macro-anchoring mechanism (A1) severely degrades the reconstruction quality (MAE increases from 2.19 to 8.56, and the missing-region $R^{2}$ drops to −9.65) and yields the worst downstream RUL accuracy among the GAN variants (17.45). Without the stage-conditioning anchor, the generator loses the lifecycle-position prior and can no longer align the synthesized dynamics with the correct degradation stage, which confirms that macro-anchoring is a key component. Second, the encoder-free variant (A2) attains an even lower pointwise reconstruction error (1.69) than the full model. This apparent advantage, however, stems from an expensive multi-start random search over the latent space that tightly fits the observed context: it provides no downstream benefit (RUL MAE 17.10 versus 15.85 for the full model) and requires far more inference time than the single-shot encoder initialization. The encoder is therefore justified by its efficiency and its balanced reconstruction–prognosis trade-off rather than by raw pointwise accuracy alone. The physics-free configuration is analyzed separately in item (4) through the term-wise and aggregate physics ablation (Table 9 and Table 11). To visually examine the degradation of imputation quality, Figure 9 compares the repaired trajectories of the variants for a typical missing segment. As shown, the full model closely tracks the true degradation trajectory. In contrast, A1 (without macro-anchoring) deviates markedly from the true trajectory, visually illustrating the stage-misalignment associated with its strongly negative reconstruction $R^{2}$: the unconditioned generator exhibits mode-collapse-like behavior. A2 (without Encoder) exhibits noticeable drift in fine-grained capacity variations. The physics-free configuration displays high-frequency oscillations around the degradation trend, consistent with its 5–10-fold inflated first-order fluctuation level. This behavior indicates that the physics loss acts as a useful prior; without it, the model explores regions that deviate from the expected degradation behavior, reducing the sample efficiency needed to generalize on a limited 9-cell dataset.

(4) Physics-constraint term-wise ablation and weight sensitivity

The module-level ablation above validates each architectural component. To quantify the individual contribution of each degradation-informed physical term in $L_{\mathrm{hybrid}}$, we further conduct term-wise ablation: w/o $L_{\mathrm{mag}}$, w/o $L_{\mathrm{shape}}$ (jointly removing the first- and second-order terms, whose individual removals are also reported), and w/o $L_{\mathrm{var}}$, together with w/o all physics terms ($\lambda_{\mathrm{hyb}}=0$). All variants are retrained under identical configurations (training cells {2–9}, 500 epochs) and evaluated on the held-out Cell #1 with ten masked windows under the same protocol as Section 4.5. Table 9 reports both the reconstruction fidelity on the missing region and the downstream RUL accuracy.

The results support a clear division of roles among the three physical terms. First, $L_{\mathrm{mag}}$ is indispensable for training stability: removing it causes the adversarial game to collapse (the discriminator loss vanishes and the generator objective diverges during training), which degrades both layers catastrophically. Second, the first-order term contributes most to reconstruction fidelity among the individual terms: removing it alone raises the reconstruction error noticeably and flips the missing-region $R^{2}$ from +0.44 to −0.08, indicating that fade-rate consistency is the dominant shape prior. Third, $L_{\mathrm{var}}$ and the second-order term provide smaller but consistent fidelity gains. Finally, removing all physics terms jointly ($\lambda_{\mathrm{hyb}}=0$) yields the largest degradation among the non-collapsed variants, confirming that the three terms are complementary rather than redundant. It is also noted that the downstream RUL differences among the single-term variants lie within the ±1 cycle run-to-run variability of the evaluation protocol, indicating that the RUL predictor tolerates moderate reconstruction-fidelity loss; the benefits of the individual physical terms manifest primarily at the reconstruction layer, whereas their joint removal significantly harms both layers.

To examine the sensitivity of the framework to the overall physics-loss weight, $\lambda_{\mathrm{hyb}}$ is varied over {0,0.25,0.5,1.0}, where 0.5 is the current setting. Table 11 shows that both reconstruction fidelity and downstream RUL accuracy improve monotonically with $\lambda_{\mathrm{hyb}}$ and saturate beyond the current value, with only marginal further gains when the weight is doubled. The framework is therefore not critically dependent on fine-tuning this weight within [0.25,1.0], and the current setting of $\lambda_{\mathrm{hyb}}=0.5$ represents a reasonable trade-off between physics regularization strength and adversarial flexibility.

These ablation results also enable a process-level explanation of how the physical constraints influence the prognosis, rather than a mere comparison of final metrics. Removing the physics terms deteriorates the reconstruction chain step by step: the pointwise reconstruction error on the missing region first grows by 122% (MAE from 2.19 to 4.85), the missing-region $R^{2}$ simultaneously flips from +0.44 to −2.36, which reflects a systematic distortion of the degradation trend (fade-rate and curvature) rather than isolated noise, and this distorted trajectory is then propagated into the downstream predictor, raising the RUL error by 11% (15.85 → 17.63 cycles). In the extreme case of removing $L_{\mathrm{mag}}$, the degradation already emerges at the training level—the adversarial optimization itself collapses—so both the trajectory quality and the prognosis fail jointly. This chain, from physical regularization → degradation-trajectory quality → downstream RUL reliability, explains the mechanism through which the degradation-informed constraints take effect.

In summary, the qualitative and quantitative results support the design of the MAP-GAN framework: together, macro-anchoring and degradation-informed regularization improve reconstruction robustness and support accurate RUL prediction under continuous block missingness.

## 5. Conclusions

This paper focuses on addressing reduced RUL prediction accuracy caused by continuous block missing data in industrial equipment PHM. The main contributions are summarized as follows. The proposed framework is developed for two-stage missing sequence reconstruction and uncertainty-aware RUL prediction. The generative process is confined to a localized optimal receptive field with a degradation stage anchoring to reduce zero-padding artifacts and dimensionality contamination. Degradation-informed physical constraints are embedded into the adversarial objective to alleviate over-smoothing bias and encourage physically consistent synthesized trajectories. An encoder-guided latent space inversion mechanism is developed to capture instance-specific heterogeneous degradation signatures. An Attention-BiLSTM network integrated with Monte Carlo Dropout is constructed to quantify predictor-side epistemic uncertainty conditional on the reconstructed inputs and to provide uncertainty-aware RUL estimates. Experimental results on the lithium-ion battery dataset indicate the effectiveness of the proposed framework. Under the main 60% interior block-missing setting, the framework achieves an RUL MAE numerically comparable to the complete-data reference (11.57±9.65 versus 11.40±9.86 cycles), reduces the RUL error by approximately 21% relative to the classic conditional-GAN baseline, and remains within 2.2 cycles of the reference across all five evaluated missing patterns, whereas the strongest baselines degrade substantially at one-sided missing positions; these findings are further reproduced on a second, independent battery dataset.

However, there are still several aspects for further improvement. First, the data repair model in the proposed framework is mainly designed for univariate time series. Therefore, how to generalize the proposed method to missing data imputation for more complex multivariate time series is a challenging direction that deserves further investigation. Second, although the sensitivity analysis and the external validation in Section 4.5 and Section 4.6 have examined the robustness of the framework to different missing ratios, missing positions, and a second battery dataset, the training strategy still assumes a fixed missing-pattern configuration per deployment. In practical industrial scenarios, missing patterns are often random and dynamically changing. One potential approach for online adaptation is to establish an adaptive missing-pattern recognition module that dynamically adjusts the repair strategy based on real-time monitoring data, whose missing-pattern information can be obtained online. Third, the current evaluation covers two lithium-ion battery datasets (nine cells under LOOCV and fourteen cells under random splits); validating the framework on larger-scale datasets collected under diverse operating conditions and equipment types constitutes an important direction for future research.

## Figures and Tables

**Figure 1:**
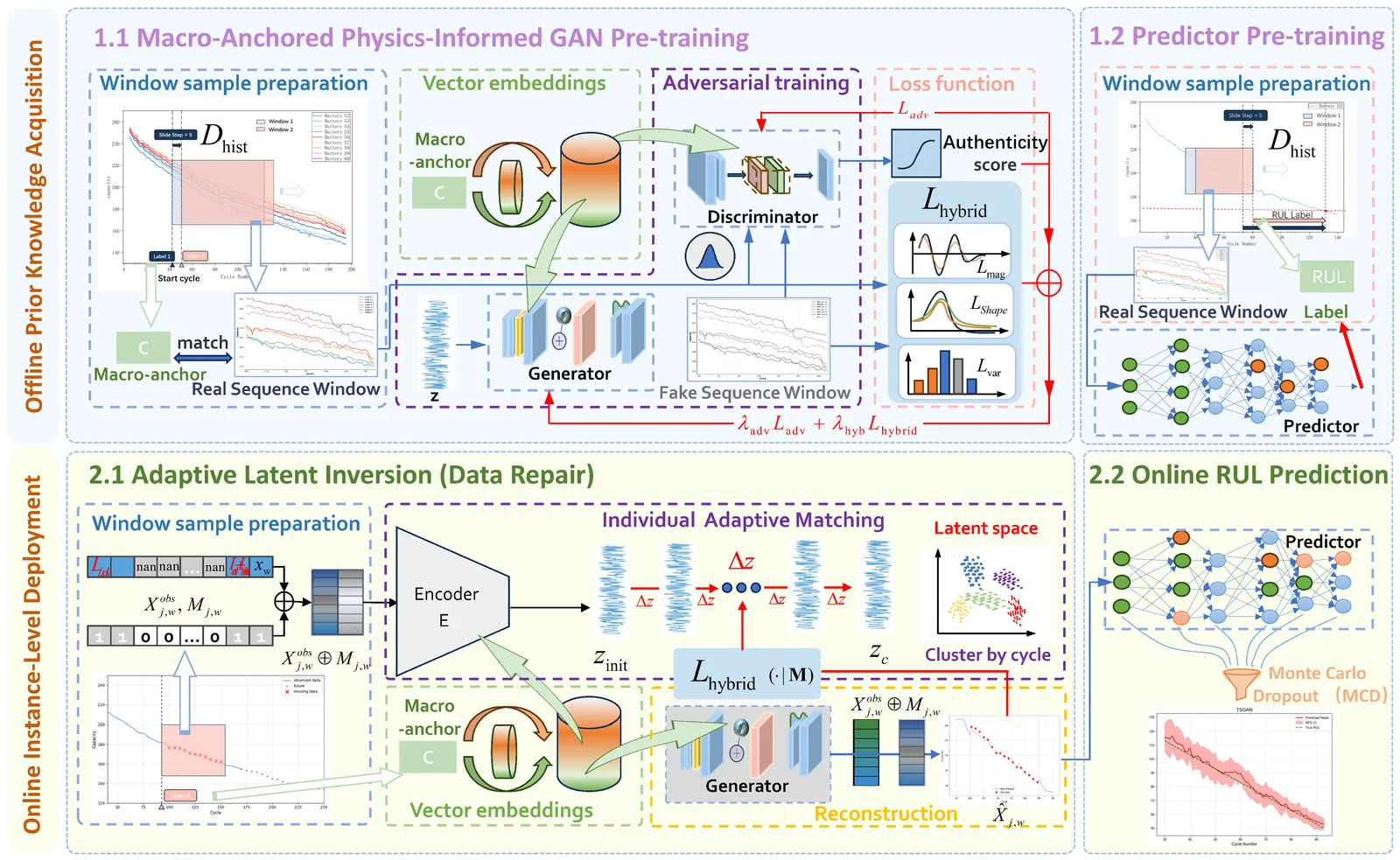
Overall architecture of the proposed MAP-GAN framework.

**Figure 2:**
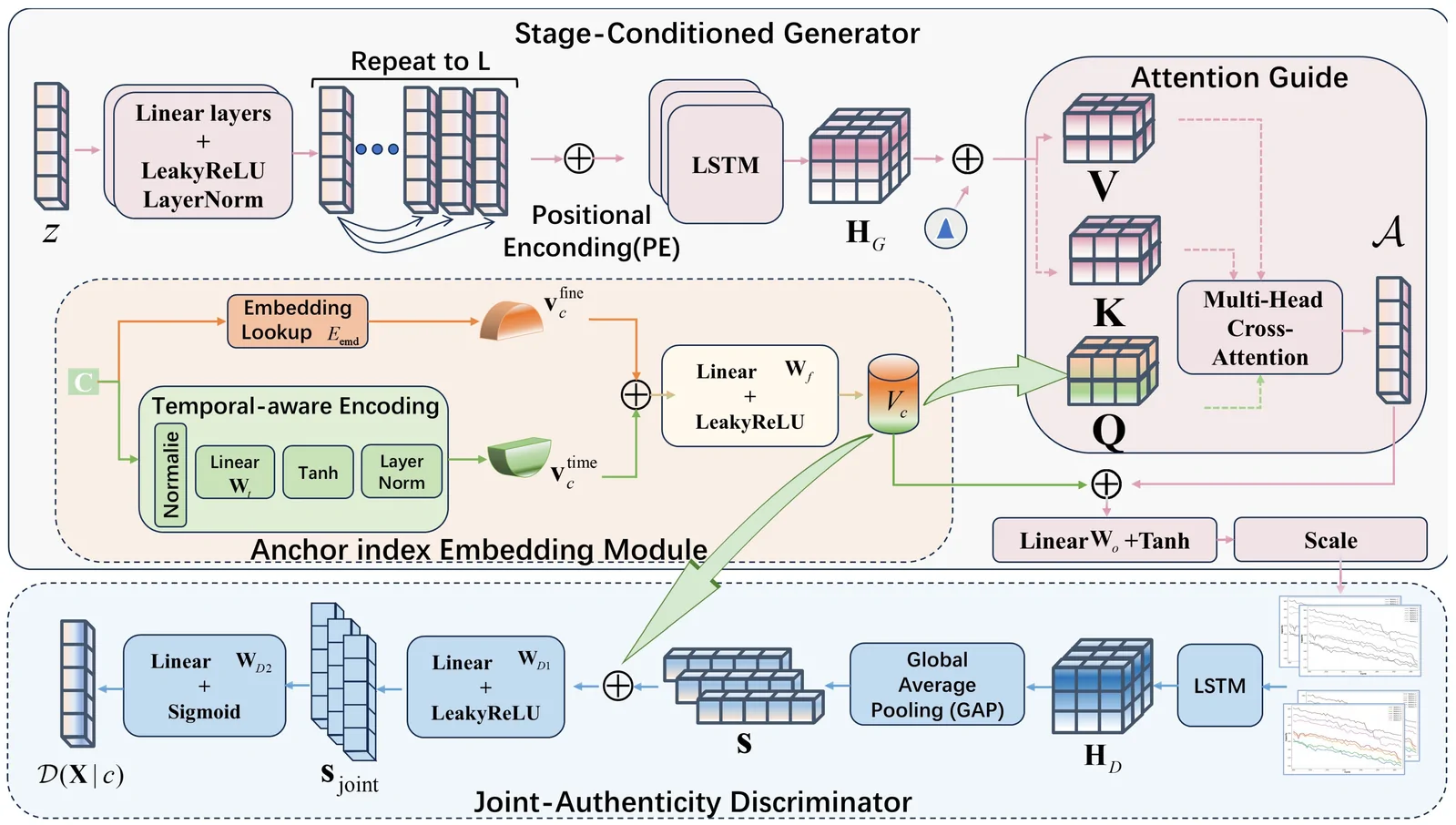
Architecture of the proposed MAP-GAN.

**Figure 3:**
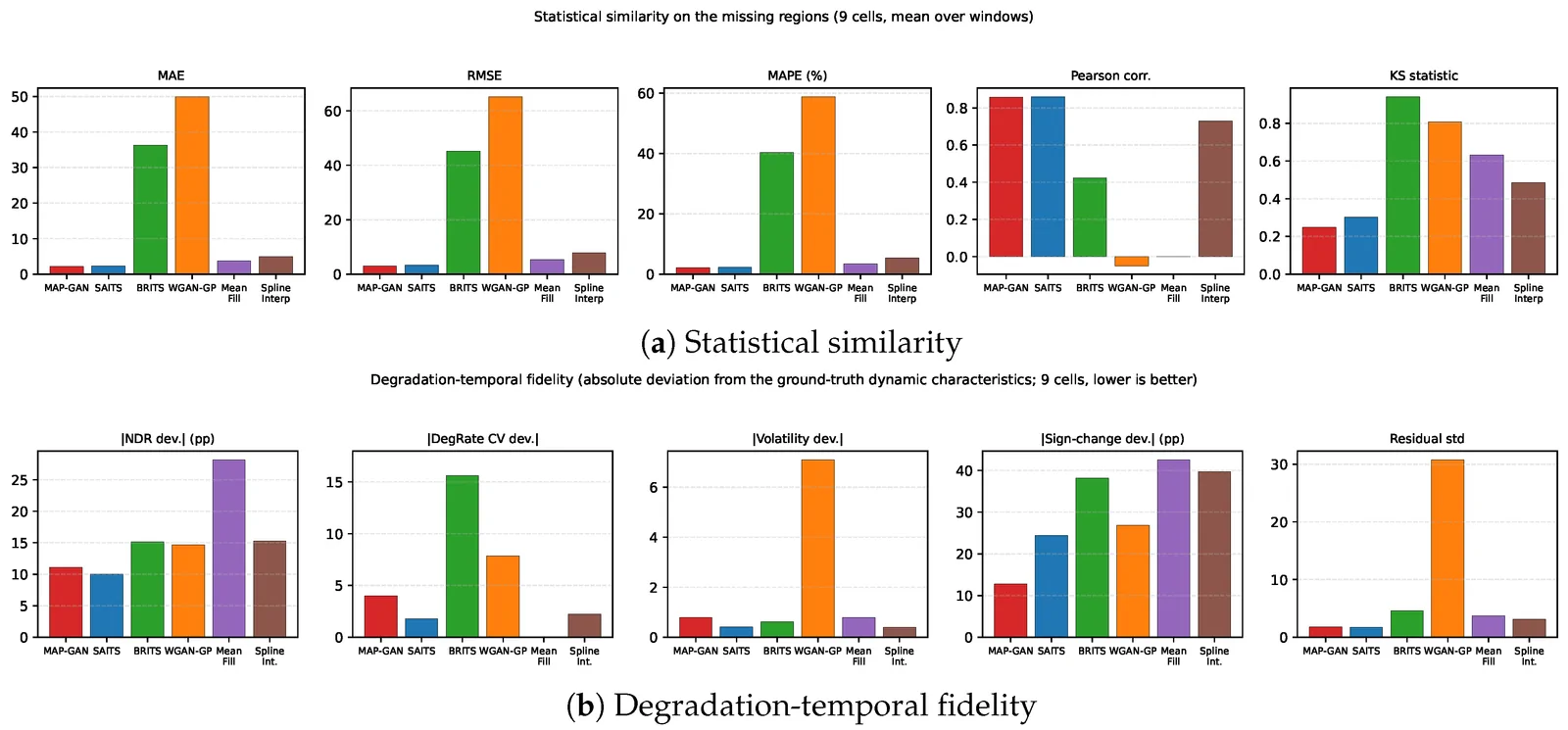
Imputation performance comparison.

**Figure 4:**
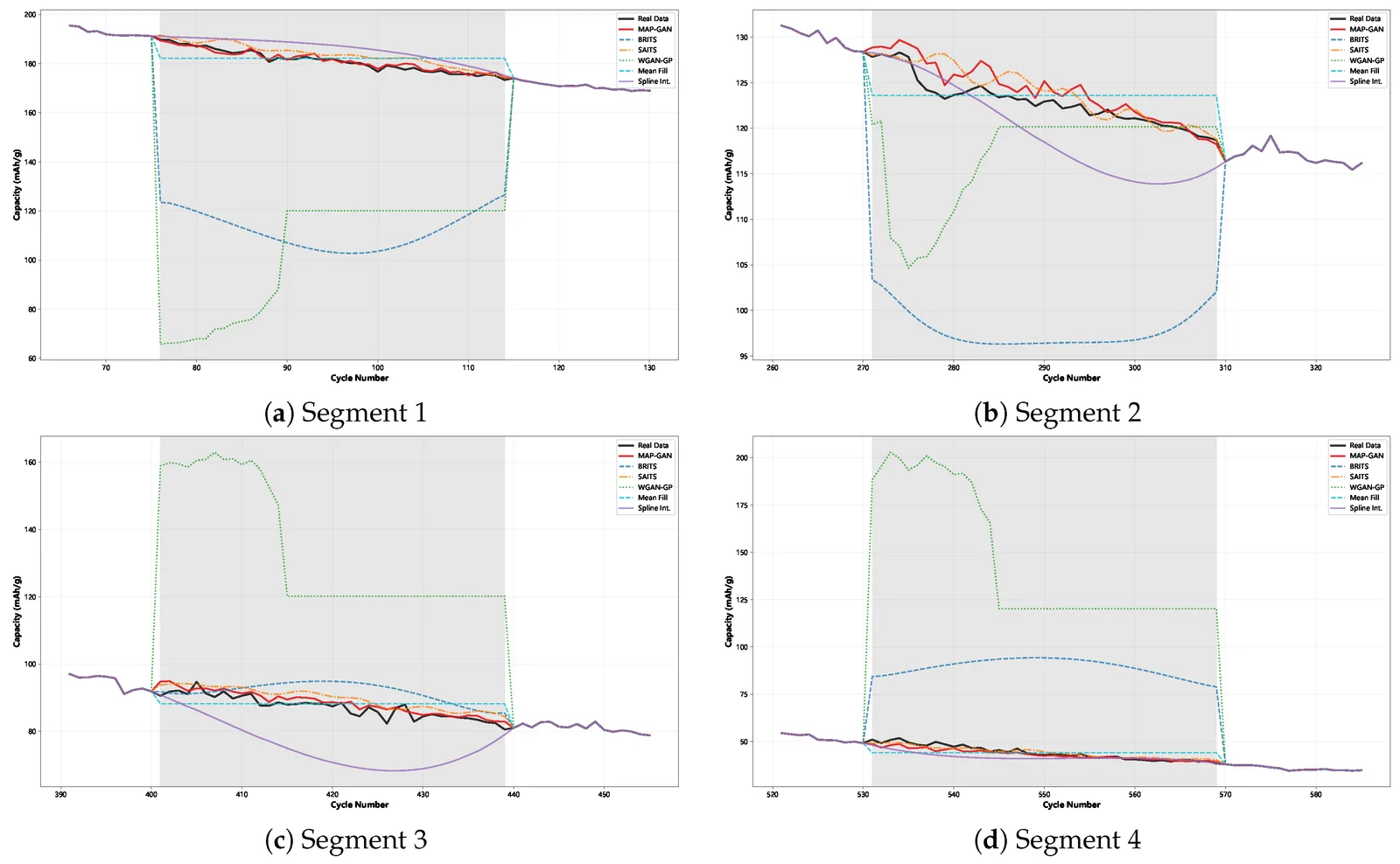
Reconstructed degradation trajectories for four representative lifecycle segments.

**Figure 5:**
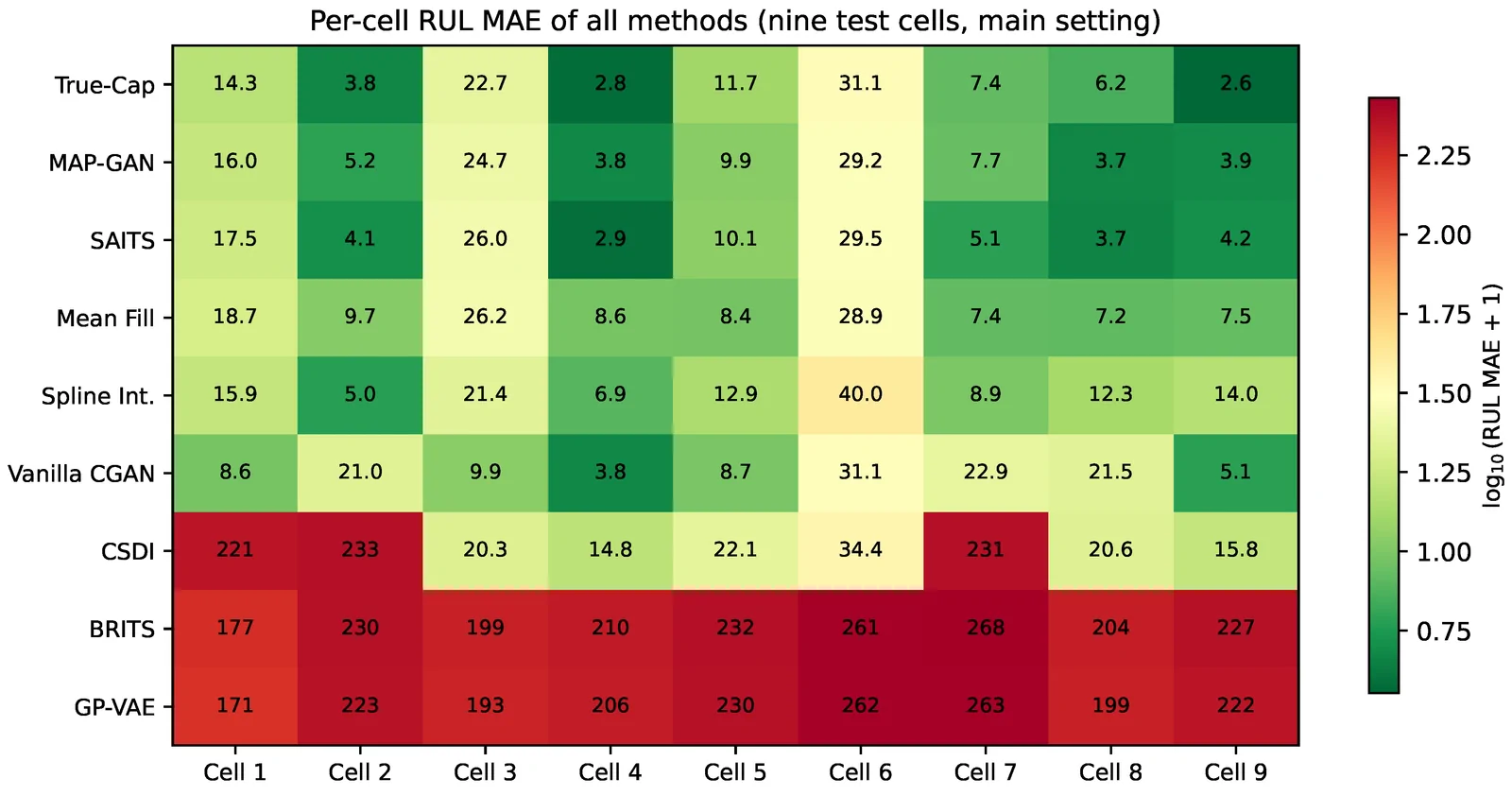
Per-cell RUL MAE across the nine test cells.

**Figure 6:**
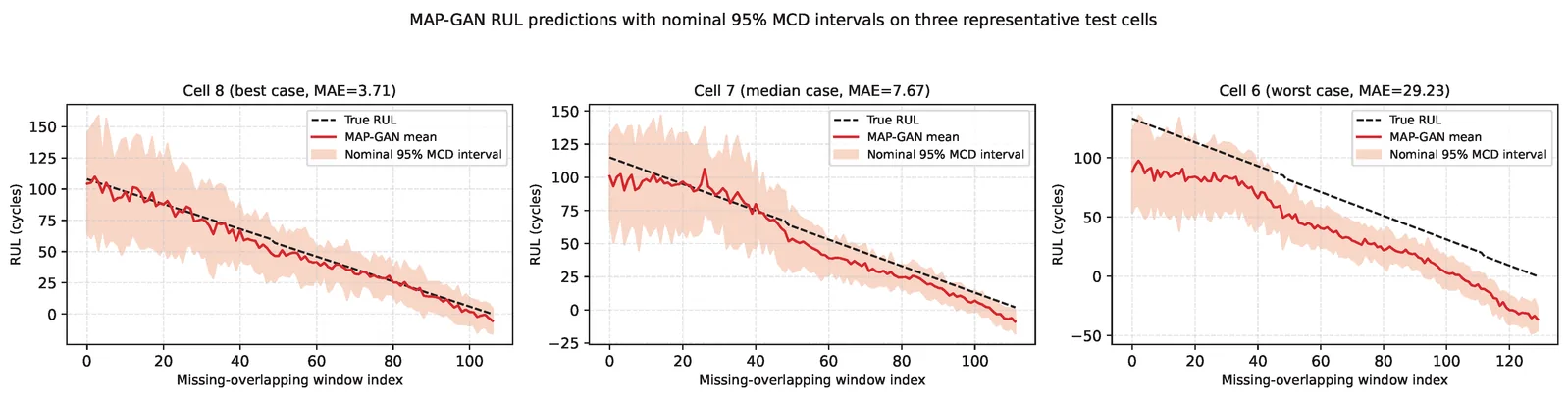
MAP-GAN RUL predictions with nominal 95% MCD intervals on representative test cells.

**Figure 7:**
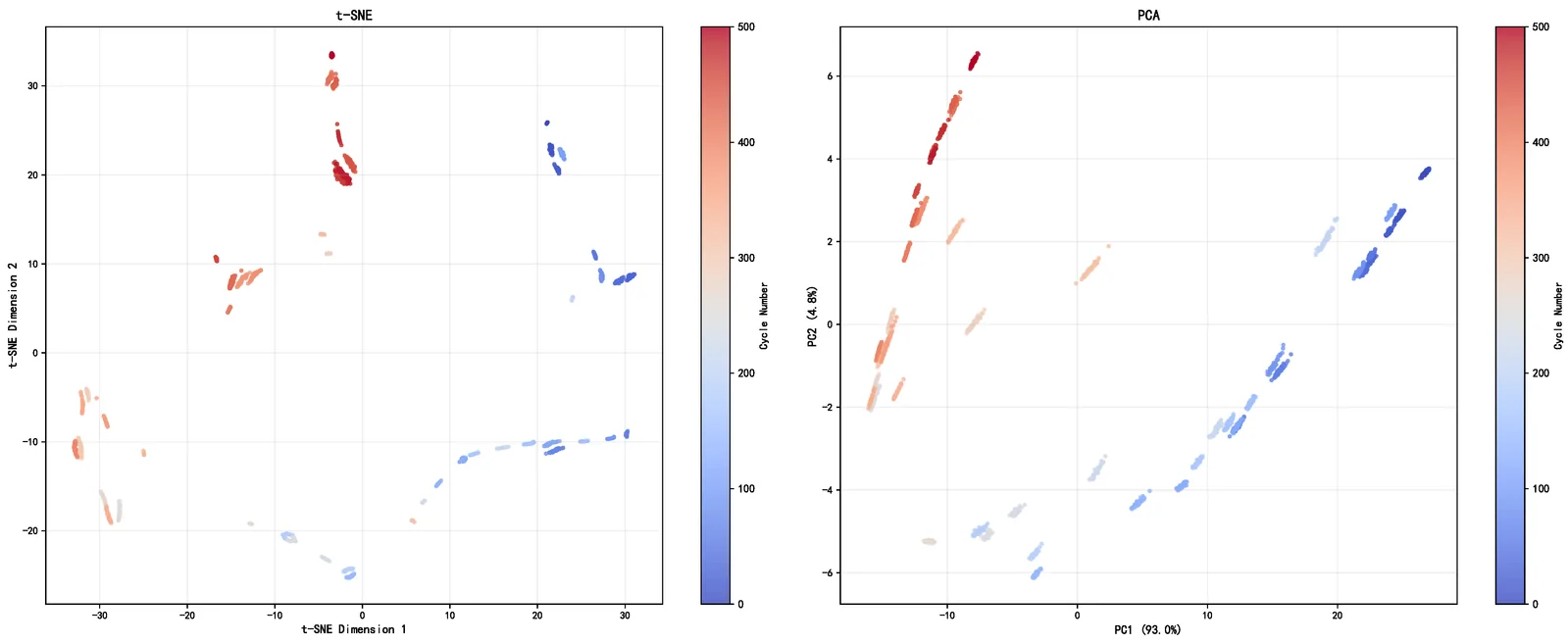
Latent-space structure of the MAP-GAN generator.

**Figure 8:**
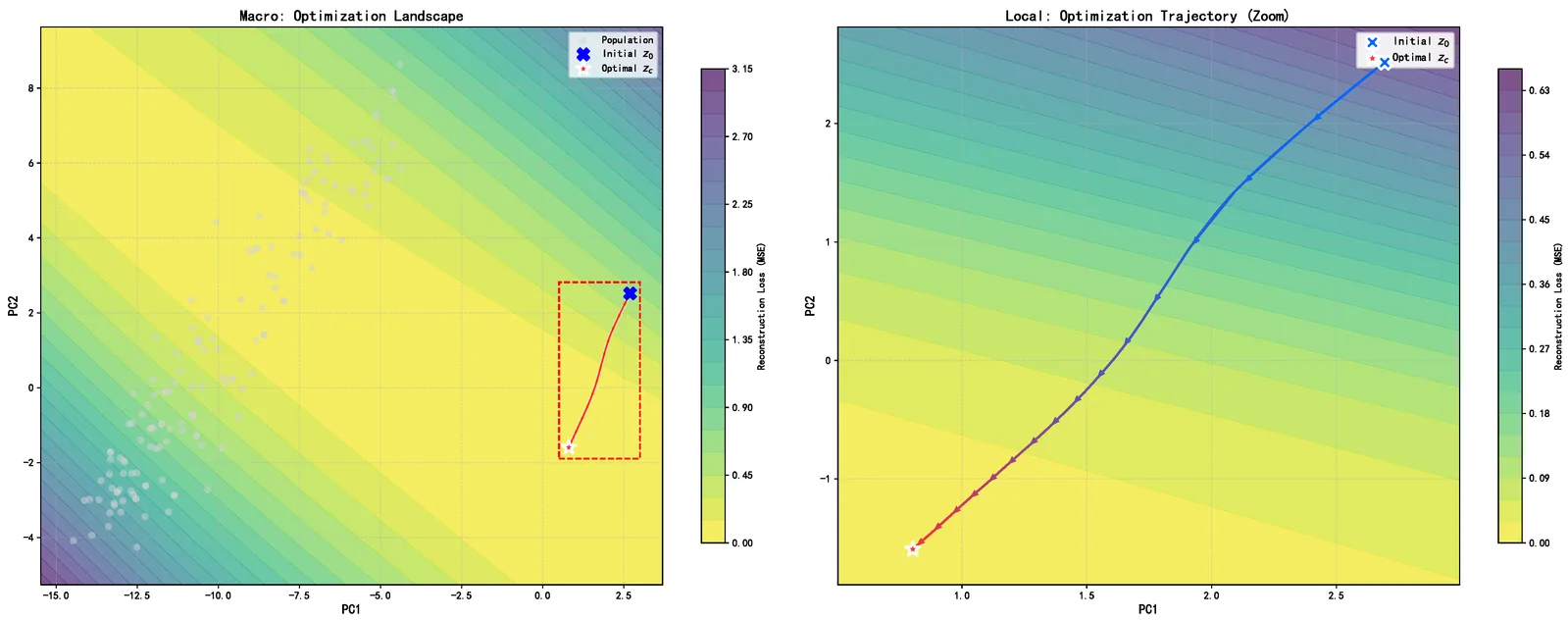
Latent-space loss landscape and inversion trajectory. The red frame and red/blue lines denote the searching historical path within the latent space.

**Figure 9:**
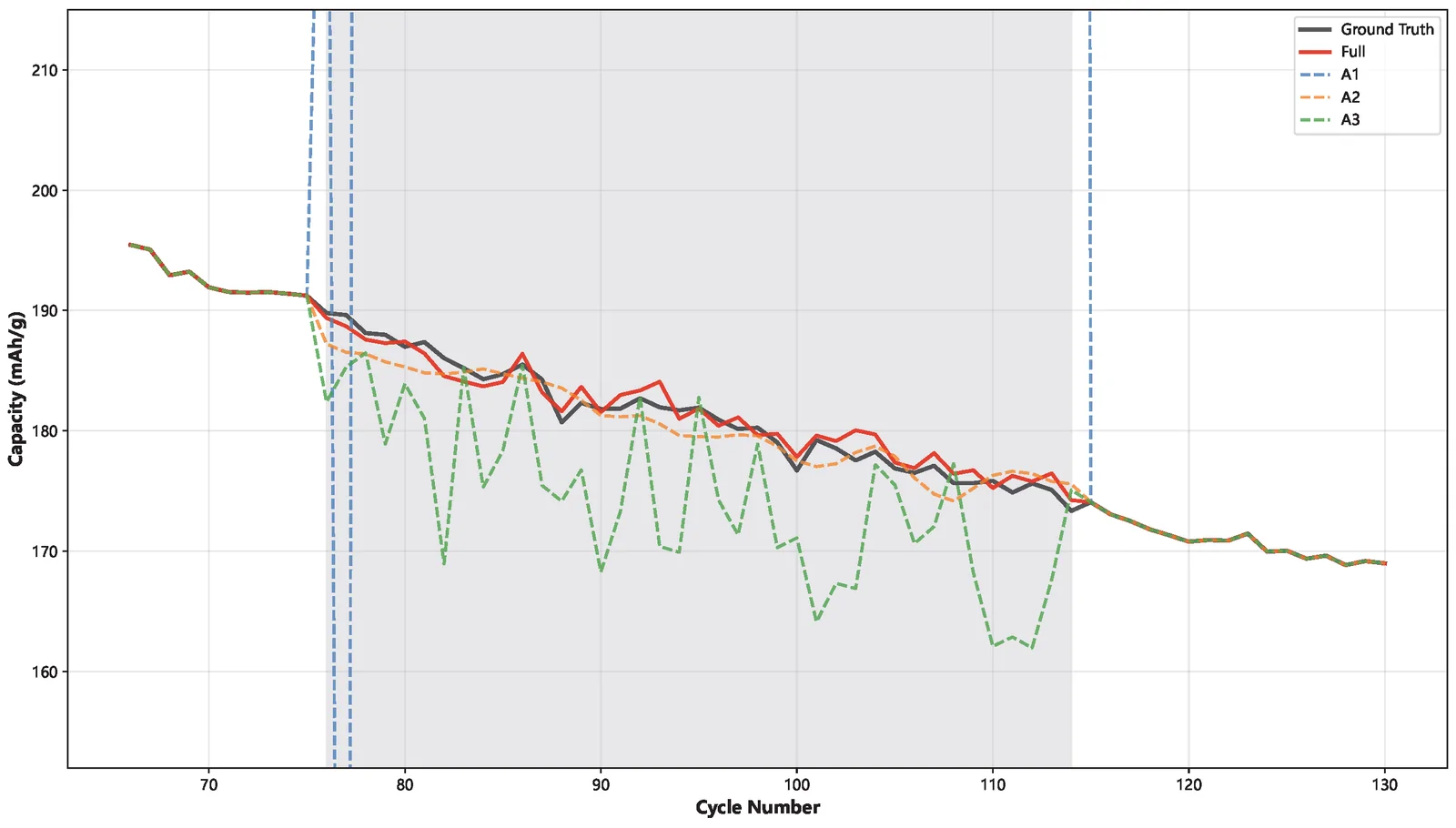
Imputation trajectories of the full model and ablation variants.

**Table 1:** Hyperparameter settings of the proposed framework.

Module	Hyperparameter	Value
Discriminator	LSTM Hidden Dim.	128
	Cycle Embedding Dim.	64
	Num. LSTM Layers	1
Encoder	Input Dim.	2
	Hidden Dim.	128
	Latent Dim.	100
BiLSTM-Attention	Sliding Window Size	25
	Num. Hidden Layers	2
	Neurons per Layer	64
	Dropout Rate	0.3
	FC Layer Structure	128-64-1
	Activation Function	ReLU
MC Dropout	Dropout Rate	0.3
	Num. MC Samplings	20
Enhanced Generator	Noise Dim.	100
	Cycle Embedding Dim.	64
	LSTM Hidden Dim.	128
	Num. LSTM Layers	2
	Dropout Probability	0.3
	Init. Noise Std. Dev.	0.1

**Table 2:** Model complexity and computational cost.

Model	Trainable Params	Per-Window Reconstruction [s]	Typical Training Time/Fold
MAP-GAN (generator)	546,945	2.43 (200 latent steps)	≈2–5 min (500 epochs)
Attention-BiLSTM-MCD	158,594	0.0011 (20 MCD passes)	<1 min (100 epochs)
SAITS	1,318,471	0.007	≈2 min (patience-terminated)
BRITS	35,216	0.347	≈11 min (patience-terminated)
CSDI	544,521	0.209	≈2 min (patience-terminated)
GP-VAE	16,993	0.004	<1 min (patience-terminated)

**Table 3:** Evaluation metrics for missing-data imputation.

Metric	Formula	Optimal
Statistical Similarity		
MAE	$\frac{1}{N_{\mathrm{miss}}}\sum_{t\in \Omega^{\mathrm{miss}}}\left|{\hat{x}}_{t}-x_{t}\right|$	Lower
RMSE	$\sqrt{\frac{1}{N_{\mathrm{miss}}}\sum_{t\in \Omega^{\mathrm{miss}}}{\left({\hat{x}}_{t}-x_{t}\right)}^{2}}$	Lower
MAPE	$\frac{1}{N_{\mathrm{miss}}}\sum_{t\in \Omega^{\mathrm{miss}}}\left|\frac{{\hat{x}}_{t}-x_{t}}{x_{t}}\right|$	Lower
Pearson *r*	$\frac{\mathrm{Cov}(\hat{X},X)}{\sigma_{\hat{X}}\sigma_{X}}$	Higher
KS	$\underset{u}{\sup}\left|\hat{F}\left(u\right)-F\left(u\right)\right|$	Lower
Degradation and Temporal Fidelity		
$E_{\mathrm{NDR}}$	$\left|\mathrm{NDR}\left(\hat{X}\right)-\mathrm{NDR}\left(X\right)\right|$	Lower
$E_{\mathrm{CV}}$	$\left|\mathrm{CV}\left(\Delta \hat{X}\right)-\mathrm{CV}\left(\Delta X\right)\right|$	Lower
$E_{\mathrm{res}}$	${\mathrm{Std}}_{t\in \Omega^{\mathrm{miss}}}\left({\hat{x}}_{t}-x_{t}\right)$	Lower
$E_{\mathrm{Vol}}$	$\left|\mathrm{Std}\left(\Delta \hat{X}\right)-\mathrm{Std}\left(\Delta X\right)\right|$	Lower
$E_{\mathrm{SCR}}$	$\left|\mathrm{SCR}\left(\Delta \hat{X}\right)-\mathrm{SCR}\left(\Delta X\right)\right|$	Lower

**Table 4:** Evaluation metrics for RUL prediction.

Metric	Formula	Optimal
**Prediction Accuracy**		
MAE	$\frac{1}{N_{\mathrm{eval}}}\sum_{n=1}^{N_{\mathrm{eval}}}\left|y_{n}-{\hat{y}}_{n}\right|$	Lower
RMSE	$\sqrt{\frac{1}{N_{\mathrm{eval}}}\sum_{n=1}^{N_{\mathrm{eval}}}{\left(y_{n}-{\hat{y}}_{n}\right)}^{2}}$	Lower
SMAPE	$\frac{1}{N_{\mathrm{eval}}}\sum_{n=1}^{N_{\mathrm{eval}}}\frac{2|y_{n}-{\hat{y}}_{n}|}{|y_{n}\left|+\right|{\hat{y}}_{n}|}$	Lower
$R^{2}$	$1-\frac{\sum_{n=1}^{N_{\mathrm{eval}}}{\left(y_{n}-{\hat{y}}_{n}\right)}^{2}}{\sum_{n=1}^{N_{\mathrm{eval}}}{\left(y_{n}-\bar{y}\right)}^{2}}$	Higher
**Uncertainty Quantification**		
CP (%)	$\frac{100}{N_{\mathrm{eval}}}\sum_{n=1}^{N_{\mathrm{eval}}}I\left({\hat{y}}_{n}^{L}\leq y_{n}\leq {\hat{y}}_{n}^{U}\right)$	Target 100γ
PI_Width	$\frac{1}{N_{\mathrm{eval}}}\sum_{n=1}^{N_{\mathrm{eval}}}\left({\hat{y}}_{n}^{U}-{\hat{y}}_{n}^{L}\right)$	Lower *
CWC	$\mathrm{NMPIW}\;\left(1+e^{-\eta (\mathrm{CP}/100-\gamma )}\right)$	Lower

Here, $N_{\mathrm{eval}}$ is the number of evaluated points, $y_{n}$ and ${\hat{y}}_{n}$ denote the ground-truth and predicted RUL at evaluation point *n*, $\left[{\hat{y}}_{n}^{L},{\hat{y}}_{n}^{U}\right]$ is the predictive interval, γ is the nominal coverage, and $\bar{y}$ is the mean of the ground-truth labels. * Lower values indicate better performance when acceptable prediction-interval coverage is maintained.

**Table 5:** Local imputation performance on four segments of Cell #1.

Metric	Segment 1						Segment 2					
	MAP-GAN	SAITS	BRITS	WGAN-GP	Mean Fill	Spline Int.	MAP-GAN	SAITS	BRITS	WGAN-GP	Mean Fill	Spline Int.
Statistical Similarity												
MAE	4.124	6.315	50.896	47.615	9.404	**1.316**	0.741	**0.597**	24.119	17.826	2.059	0.927
RMSE	5.780	9.212	71.612	66.552	13.441	**1.998**	1.212	**1.032**	34.246	24.924	3.139	1.461
MAPE	1.937	2.952	23.849	22.344	4.435	**0.613**	0.479	**0.386**	15.540	11.502	1.336	0.595
Pearson *r*	0.980	0.978	−0.876	−0.832	0.901	**0.995**	0.974	**0.983**	−0.827	−0.750	0.902	0.968
KS_statistic	0.333	0.410	0.513	0.513	0.513	**0.154**	0.205	**0.179**	0.513	0.513	0.513	0.256
Degradation & Temporal Fidelity												
$E_{\mathrm{NDR}}$	10.526	**0.000**	39.474	2.632	5.263	5.263	15.789	2.632	39.474	7.895	10.526	**0.000**
$E_{\mathrm{CV}}$	0.915	0.038	4.262	6.187	3.050	**0.151**	0.494	0.728	1.873	5.209	1.166	**0.479**
$E_{\mathrm{res}}$	4.049	6.708	50.377	46.498	9.603	**1.503**	1.014	**0.841**	24.312	17.420	2.369	1.129
$E_{\mathrm{Vol}}$	0.960	0.383	11.303	13.072	3.402	**0.175**	0.311	**0.184**	4.367	4.562	0.441	0.255
$E_{\mathrm{SCR}}$	13.514	**5.405**	**5.405**	**5.405**	8.108	10.811	**5.405**	**5.405**	13.514	13.514	10.811	13.514
	**Segment 3**						**Segment 4**					
	**MAP-GAN**	**SAITS**	**BRITS**	**WGAN-GP**	**Mean Fill**	**Spline Int.**	**MAP-GAN**	**SAITS**	**BRITS**	**WGAN-GP**	**Mean Fill**	**Spline Int.**
Statistical Similarity												
MAE	2.295	**0.683**	11.896	16.657	2.025	5.094	0.764	**0.586**	8.556	21.194	1.445	4.267
RMSE	3.155	**0.899**	13.041	19.489	2.575	6.836	1.408	**1.224**	13.848	34.208	2.654	7.222
MAPE	2.095	**0.628**	10.919	15.224	1.845	4.762	1.166	**0.879**	13.147	32.681	2.267	6.503
Pearson *r*	0.598	**0.961**	0.686	0.284	0.602	−0.750	0.972	**0.984**	0.919	0.855	0.950	0.150
KS_statistic	0.231	**0.179**	0.846	0.846	0.513	0.641	**0.154**	0.179	0.385	0.385	0.359	0.385
Degradation & Temporal Fidelity												
$E_{\mathrm{NDR}}$	21.053	**2.632**	**2.632**	10.526	26.316	26.316	**2.632**	**2.632**	10.526	10.526	10.526	10.526
$E_{\mathrm{CV}}$	9.041	**0.717**	1.488	36.334	2.008	>100 *	1.167	**0.016**	0.657	2.896	0.726	4.663
$E_{\mathrm{res}}$	2.900	**0.839**	5.343	10.117	2.352	4.965	1.338	**1.120**	10.889	26.852	2.286	5.826
$E_{\mathrm{Vol}}$	2.218	0.181	1.727	4.828	**0.170**	0.214	0.322	0.248	2.036	8.212	0.259	**0.032**
$E_{\mathrm{SCR}}$	**8.108**	27.027	48.649	35.135	51.351	48.649	10.811	**0.000**	10.811	8.108	8.108	10.811

Bold values indicate the best performance, including tied values. * The mean fade rate within this segment approaches zero, so the CV deviation degenerates.

**Table 6:** RUL prediction performance across the nine test cells.

Method	MAE	RMSE	$R^{2}$	CP (%)	PI_Width	CWC
True-Cap (reference)	**11.40±9.86**	**12.07±9.68**	**0.767**	77.9	42.30	1.68 × $10^{3}$
MAP-GAN (proposed)	11.57±9.65	12.49±9.38	0.746	77.0	43.99	2.67 × $10^{3}$
SAITS	11.46±10.35	12.37±10.12	0.732	**79.7**	44.64	**7.13 × $10^{2}$**
Mean Fill	13.63±8.67	15.32±8.33	0.666	75.3	44.83	6.41 × $10^{3}$
Vanilla CGAN	14.73±9.54	16.68±10.02	0.645	67.5	**39.41**	2.79 × $10^{5}$
Spline Int.	15.26±10.52	17.01±10.65	0.631	66.0	42.14	6.32 × $10^{5}$
CSDI	90.28±103.59	125.16±148.64	−41.39	48.7	75.00	6.50 × $10^{9}$
BRITS	222.98±29.43	239.29±29.91	−59.87	2.4	94.80	9.35 × $10^{19}$
GP-VAE	218.85±30.57	234.03±31.18	−57.04	2.5	92.85	8.68 × $10^{19}$

Bold values indicate the best performance.

**Table 7:** RUL MAE under different missing-data patterns.

Method	R20%	R40%	R60% (Main)	P-Early	P-Late
True-Cap (reference)	11.75	11.56	**11.40**	**11.48**	**11.44**
MAP-GAN (proposed)	11.80	**11.50**	11.57	12.62	13.63
SAITS	11.96	11.51	11.46	15.77	19.44
Mean Fill	11.72	12.16	13.63	18.02	17.13
Spline Int.	11.62	11.71	15.26	237.71	259.76
Vanilla CGAN	**11.37**	12.38	14.73	15.19	15.97
CSDI	21.84	27.69	90.28	23.96	33.02
BRITS	96.21	175.73	222.98	249.40	222.77
GP-VAE	154.91	200.11	218.85	212.24	204.29
Bold values indicate the best performance.					

**Table 8:** External validation on the HNEI dataset.

Method	R20%	R40%	R60% (Main)	P-Early	P-Late
True-Cap (reference)	32.76 ± 1.94	32.24 ± 1.56	**31.94 ± 1.38**	32.11 ± 1.48	**31.94 ± 1.49**
MAP-GAN (proposed)	**31.64 ± 1.85**	**31.67 ± 1.25**	31.97 ± 1.27	**30.43 ± 0.85**	34.74 ± 1.56
Vanilla CGAN	32.64 ± 1.76	34.18 ± 1.23	35.10 ± 1.60	31.88 ± 1.60	36.93 ± 0.49
SAITS	32.62 ± 1.11	32.47 ± 1.12	32.66 ± 1.08	36.71 ± 2.88	34.88 ± 0.55
Mean Fill	32.98 ± 1.70	32.72 ± 1.43	33.02 ± 1.30	34.51 ± 0.45	**34.56 ± 0.81**
Spline Int.	32.52 ± 1.85	33.98 ± 2.27	32.52 ± 0.93	210.14 ± 8.92	204.49 ± 11.38
BRITS	44.48 ± 2.09	69.89 ± 3.46	90.37 ± 5.97	91.30 ± 6.63	87.14 ± 5.93
CSDI	61.09 ± 35.45	76.19 ± 57.86	87.10 ± 73.04	80.16 ± 57.88	81.46 ± 58.55
GP-VAE	42.90 ± 1.31	67.13 ± 0.78	84.43 ± 0.82	83.71 ± 0.90	80.57 ± 0.54

Bold values indicate the best performance.

**Table 9:** Physics-constraint ablation results on Cell #1.

Variant	Reconstruction (Missing Region)			Downstream RUL		
	MAE	RMSE	$R^{2}$	MAE	RMSE	$R^{2}$
Full ($\lambda_{\mathrm{hyb}}=0.5$)	2.19	2.74	0.438	15.85	16.33	0.613
w/o $L_{\mathrm{mag}}$	177.76	230.26	−4946.1	35.24	45.39	−1.988
w/o $L_{\mathrm{shape}}$	2.67	3.27	0.260	15.22	15.68	0.644
w/o first-order term	2.87	3.60	−0.083	15.08	15.50	0.652
w/o second-order term	**2.09**	**2.59**	**0.490**	15.63	16.30	0.615
w/o $L_{\mathrm{var}}$	2.62	3.28	0.100	**14.87**	**15.26**	**0.662**
w/o all physics ($\lambda_{\mathrm{hyb}}=0$)	4.85	5.87	−2.360	17.63	18.31	0.514

Bold values indicate the best performance.

**Table 10:** Module-level ablation results on Cell #1.

Model	Reconstruction (Missing Region)				Downstream RUL	
	MAE	RMSE	$R^{2}$	Pearson r	MAE	$R^{2}$
Full	2.19	2.74	0.438	0.822	**15.85**	**0.613**
A1 (w/o macro-anchoring)	8.56	10.42	−9.649	0.666	17.45	0.531
A2 (w/o Encoder)	**1.69**	**1.99**	**0.740**	**0.958**	17.10	0.555

Bold values indicate the best performance.

**Table 11:** Sensitivity to the physics-loss weight ($\lambda_{\mathrm{hyb}}$).

$\lambda_{\mathrm{hyb}}$	Recon. MAE	Recon. $R^{2}$	RUL MAE	RUL $R^{2}$
0 (no physics)	4.85	−2.360	17.63	0.514
0.25 (0.5×)	2.57	0.236	16.61	0.580
0.5 (current)	2.19	0.438	15.85	0.613
1.0 (2×)	**2.04**	**0.503**	**15.48**	**0.628**

Bold values indicate the best performance, including tied values.

## Data Availability

The primary nine-cell dataset used in this study is not publicly available because the authors do not have permission to share it. The HNEI dataset used for the external validation is publicly available from the Hawaii Natural Energy Institute via the Battery Archive repository (https://batteryarchive.org accessed on 2 July 2026).

## Abbreviations and Nomenclature

The following abbreviations and nomenclature are used in this manuscript:

GAN	Generative Adversarial Network
MAP-GAN	Macro-Anchored Physics-Informed GAN
RUL	Remaining Useful Life
PHM	Prognostics and Health Management
CM	Condition Monitoring
IIoT	Industrial Internet of Things
CNN	Convolutional Neural Network
RNN	Recurrent Neural Network
LSTM	Long Short-Term Memory
BiLSTM	Bidirectional LSTM
TCN	Temporal Convolutional Network
DNN	Deep Neural Network
DCGAN	Deep Convolutional GAN
CSDI	Conditional Score-based Diffusion Imputation
GP-VAE	Gaussian Process Variational Autoencoder
HNEI	Hawaii Natural Energy Institute
MCD	Monte Carlo Dropout
MCAR	Missing Completely at Random
EOL	End of Life
CC	Constant Current
LOOCV	Leave-One-Out Cross-Validation
MSE	Mean Squared Error
MAE	Mean Absolute Error
RMSE	Root Mean Squared Error
MAPE	Mean Absolute Percentage Error
SMAPE	Symmetric MAPE
KS	Kolmogorov–Smirnov
NDR	Negative Difference Ratio
CV	Coefficient of Variation
CWC	Coverage Width-based Criterion
CP	Coverage Probability
CI	Confidence Interval (used only for bootstrap intervals)
PI	Prediction Interval
NMPIW	Normalized Mean Prediction Interval Width
Indices and sizes	
*i*, *j*	index of a historical (run-to-failure) unit; index of an online (in-service) unit
*t*, *b*, *c*, *s*	operating-cycle index; mini-batch index; macro-anchor index; Monte Carlo Dropout pass index
*n*	aggregate evaluation-point index used in the metric definitions
*N*, $N_{\mathrm{online}}$, $N_{\mathrm{eval}}$	number of historical units; number of online units; number of evaluated RUL points
$T_{i}$, *B*, *L*, *W*, *S*	lifecycle length; mini-batch size; generator micro-window length; RUL-predictor window length; number of stochastic forward passes
Datasets, sequences, and masks	
$D_{\mathrm{hist}}$, $D_{\mathrm{online}}$	complete offline historical dataset; incomplete online monitoring dataset
$X_{i}^{\mathrm{hist}}$, $y_{i}^{\mathrm{hist}}$	full-lifecycle degradation sequence and cycle-wise RUL-label sequence of historical unit *i*
$X_{j}^{\mathrm{true}}$, $X_{j}^{\mathrm{online}}$	latent noise-free degradation trajectory; fully sampled noisy sensing sequence of online unit *j*
$X_{j}^{\mathrm{obs}}$, $X_{j}^{\mathrm{miss}}$	observed and missing parts induced by the binary mask
$M_{j}$	binary mask vector ($m_{j,t}=1$: observed; $m_{j,t}=0$: missing)
$\Omega_{j}^{\mathrm{obs}}$, $\Omega_{j}^{\mathrm{miss}}$	sets of observed and missing cycle indices of online unit *j*
${\hat{X}}_{j}$, ${\hat{x}}_{j,t}$	reconstructed full sequence and its value at cycle *t*
Δ, $\Delta^{2}$	first- and second-order forward temporal-difference operators

Networks and latent codes	
$G_{\psi }$, $D_{\phi }$	stage-conditioned generator and joint-authenticity discriminator
$E_{\omega }$, $f_{\theta }$	inversion encoder and RUL predictor
z, $z_{j,c}^{\left(0\right)}$, $z_{j,c}^{⋆}$	Gaussian latent code; encoder initialization; instance-optimized latent code for online unit *j* at anchor *c*
Losses and physical operator	
$L_{\mathrm{adv}}$, $L_{\mathrm{hybrid}}$, $L_{\mathrm{inv}}$	adversarial loss; degradation-informed hybrid loss; masked latent-inversion loss
$L_{\mathrm{mag}}$, $L_{\mathrm{shape}}$, $L_{\mathrm{var}}$	amplitude, multi-order shape, and fluctuation-variance terms
$\lambda_{\mathrm{adv}}$, $\lambda_{\mathrm{hyb}}$, $\lambda_{\mathrm{mag}}$, $\lambda_{\mathrm{shape}}$, $\lambda_{\mathrm{var}}$	weights of the corresponding loss terms
$P_{\varepsilon_{1}}\left(\cdot \right)$	physical boundary projection operator with bound $\varepsilon_{1}$
Prognostic uncertainty	
${\hat{y}}_{j,t}^{\left(s\right)}$	RUL prediction at cycle *t* of online unit *j* in stochastic pass *s*
${\hat{\mu }}_{j,t}$, ${\hat{\sigma }}_{j,t}$	Monte Carlo predictive mean and standard deviation
β, $z_{1-\beta /2}$	interval miscoverage probability and corresponding standard-normal quantile
γ, η	nominal coverage probability (γ=1−β); penalty coefficient of the CWC metric
